# Nipah virus matrix protein uses cortical actin to stabilize the virus assembly sites and promote budding

**DOI:** 10.1126/sciadv.adw4609

**Published:** 2025-09-19

**Authors:** Jingjing Wang, Vicky Kliemke, Mengyu Zhang, Jinxin Liu, Giuliana Leonarda Matta, Qian Wang, Yuhang Luo, GuanQun Liu, Qian Liu

**Affiliations:** ^1^ Institute of Parasitology, Faculty of Agricultural and Environmental Sciences, McGill University, Sainte-Anne-de-Bellevue, Quebec, Canada.; ^2^ Mark Wainberg Center for Viral Diseases, Lady Davis Institute, Montreal, Quebec, Canada.; ^3^ Department of Microbiology and Immunology, Faculty of Medicine and Health Sciences, McGill University, Montreal, Quebec, Canada.; ^4^ The McGill Research Center on Complex Traits, McGill University, Montreal, Quebec, Canada.

## Abstract

Several enveloped viruses, including paramyxoviruses, assemble and bud from the host plasma membrane (PM). Nipah virus (NiV), a deadly zoonotic paramyxovirus, uses its matrix protein (M) to drive virus assembly and budding through dimerization and PM interaction. We show that NiV-M–mediated virus-like particle (VLP) production depends on its interaction with host F-actin via its carboxyl-terminal domain. We demonstrate that F-actin retains NiV-M assembly sites at the PM by analyzing NiV-M assembly kinetics. Disrupting actin dynamics or NiV-M–actin interaction alters M nanoscale organization and reduces membrane retention, without affecting initial recruitment. We also show that the Arp2/3 complex, an actin-branching factor, promotes VLP production. Inhibiting Arp2/3 reduces NiV-M retention at the PM and impairs protrusion formation while leaving the assembly rate unchanged. These findings suggest that the host F-actin retains NiV assembly sites on the PM and promotes virus budding via Arp2/3-driven actin branching.

## Teaser

Nipah virus matrix protein uses cortical actin to anchor assembly sites and drive budding.

## INTRODUCTION

Paramyxoviruses are a group of clinically important viruses that are mainly transmitted via respiratory droplets and direct contact (*1*, *2*). Measles (MeV) and human parainfluenza viruses (PIV) are established human pathogens and cause infections in all age groups and hospitalization every year (*3*, *4*). Nipah (NiV) and Hendra (HeV) viruses in the *Henipavirus* genus are known zoonotic viruses that cause yearly outbreaks in Southeast Asia and Australia, with more than 70% mortality rate in humans (*5*, *6*). They are capable of animal-animal, animal-human, and human-human transmissions (*5*, *6*). Despite these threats, no vaccines or therapeutics are approved for humans against these emerging paramyxoviruses (*6*).

Paramyxoviruses are enveloped, negative-strand, nonsegmented RNA viruses (*1*). The RNA genome codes for six proteins, including two membrane glycoproteins for virus entry—the fusion protein (F) and the attachment protein (H/HN/G), the matrix protein (M), and three proteins for ribonucleoprotein (RNP) complex formation and RNA genome replication—the nucleoprotein (N), the large polymerase protein (L), and the phosphoprotein (P) (*1*). Among them, M orchestrates the assembly and budding of most paramyxoviruses and, thus, plays a central role in virus transmission among host cells (*1*). A cryo–electron tomography study reveals that MeV-M protein forms two-dimensional arrays on the inner leaflet of the plasma membranes (PMs) at the MeV assembly and budding sites in infected cells and cell-free virus particles, similar to that of Newcastle disease viruses (NDV) (*7*, *8*). MeV-M proteins have also been observed to coat RNPs but dissociate with the viral membranes (*9*). X-ray crystallography has shown that the paramyxovirus M forms dimers and associates with PM by interacting with membrane lipids, phosphatidylserine (PS), and phosphatidylinositol 4,5-biphosphate [PI(4,5)P_2_], in the inner leaflet (*7*, *10*). Binding to PS and PI(4,5)P_2_ triggers conformational and electrostatic changes in M for membrane curvature generation (*10*), although the involvement of host factors in this process remains unclear.

The actin cytoskeleton consists of actin filaments (F-actin) organized into higher-order arrays capable of dynamic remodeling (*11*). Among them, the actin cortex is defined as an actin network underneath the PM (*11*). Many intracellular pathogens co-opt the host cortical actin network to fulfill the life cycles (*12*–*15*). In paramyxoviruses, MeV production is partially inhibited by actin polymerization inhibitors or stabilizers as they affect the intracellular trafficking of RNPs to the PM and virion maturation (*16*). MeV was observed to bud from microvillus-like structures where the negative-directed actin filaments are tightly associated with the budding virions (*17*). The M proteins of Sendai virus (SeV) and NDV have been shown to interact with actin filaments directly (*18*). Further, HIV-1 manipulates the actin cytoskeleton for virus production by directly interacting with the F-actin via Gag, and the HIV-1 Gag particle release from CD4 T cells depends on the Rac1-IRSp53-Wave2-Arp2/3 signaling pathway (*19*). The Arp2/3 complex nucleates the formation of branched actin, essential for the actin cortex and most filopodia (*20*, *21*). Respiratory syncytial virus requires Arp2 for the production of infectious progeny virions, and the Arp2-dependent filopodia formation and cell motility facilitate cell-to-cell virus spread (*22*). The Arp2/3-mediated actin branching prevents HIV-1 viral particle production in infected T cells, partly correlated with a decreased number of HIV-1 Gag clusters on the T cell membrane (*23*). Nonetheless, Arp2/3 and cofilin were identified as cellular binding partners of NiV-M in a proteomics-based interaction study (*24*).

Here, we show that NiV-M directly interacts with actin, which is important in maintaining the nanoscale NiV-M assembly sites at the PM and virus-like particle (VLP) production. Analysis of assembly kinetics reveals that NiV-M assembly sites transit through three stages at the PM: assembly, membrane retention, and release. The duration of membrane retention is sensitive to NiV-M-actin interaction and Arp2/3-mediated actin branching. Additionally, the Arp2/3 complex-mediated actin branching facilitates the generation of membrane protrusion and VLP production. Together, our findings highlight the critical role of F-actin in maintaining the structural integrity of NiV-M assembly sites and their retention at the PM, as well as in promoting membrane protrusion formation required for efficient viral budding.

## RESULTS

### NiV-M interacts with actin in the host cell for VLP production

**Fig. 1.**
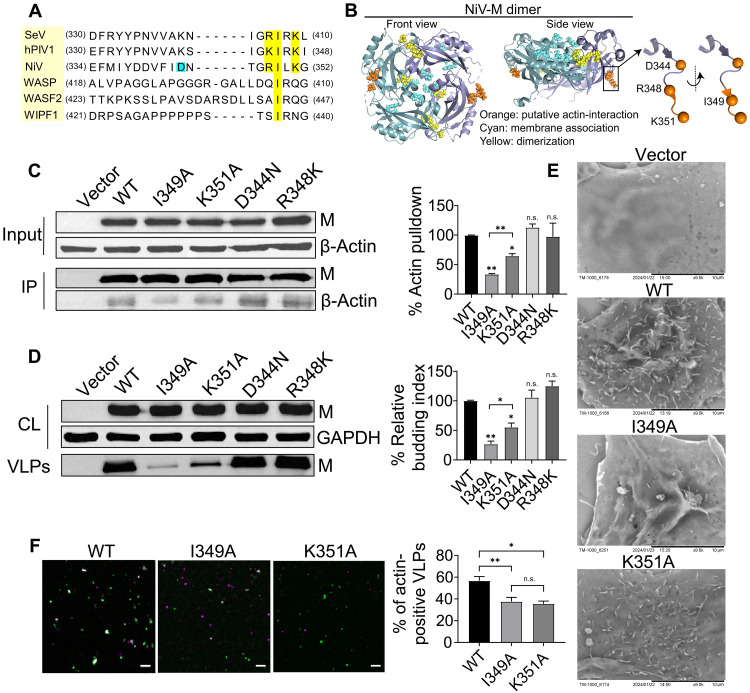
An actin-binding domain in NiV-M is key for NiV VLP production. (**A**) M protein sequences from SeV, hPIV1, and NiV, along with actin-binding proteins WASP, WASF2, and WIPF1, were aligned to identify conserved motifs. Conserved residues are in yellow, and D344 is in cyan. (**B**) Structural features of NiV-M were analyzed using the crystal structure (Protein Data Bank: 7SKT) and visualized with PyMOL. (**C**) 293T cells were transfected with 3xFLAG-tagged NiV-M constructs. β-Actin was immunoprecipitated using anti-FLAG magnetic beads. Input and IP samples were analyzed by SDS-PAGE and immunoblotting with anti-FLAG and anti–β-actin antibodies. (**D**) 293T cells were transfected with 3xFLAG-tagged NiV-M constructs or vector control. Cell lysates and VLPs were collected and analyzed by Western blot. [(C) and (D)] % Actin pulldown and relative budding index were determined based on integrated immunoblot density. (**E**) COS-7 cells stably expressing 3xFLAG-tagged NiV-M constructs were imaged by scanning electron microscopy. Scale bars, 10 μm. (**F**) VLPs (*n* > 200 per group) produced in 293T cells transfected with GFP-tagged NiV-M constructs were imaged by confocal microscopy after immunostaining with anti–β-actin and fluorescent secondary antibodies. Scale bars, 5 μm. Bars represent means ± SEM [(C) and (D)] and means ± SD (F). *P* values were obtained using one-way analysis of variance (ANOVA) with post hoc correction. Not significant (n.s.), *P* > 0.05; *P ≤ 0.05; **P ≤ 0.01. Results from ≥3 independent experiments are shown.

Previous studies show that SeV infection induces actin remodeling to promote efficient virion production by an actin-binding domain (RIRK) at the C-terminal domain of the M protein (*18*, *25*). This actin-binding domain is conserved in SeV and PIV1 and resembles the KLKK motif found in thymosin β4, which has been shown through mutational studies to be critical for actin binding (*25*). We aligned the protein sequences of NiV-M, hPIV-1-M, SeV-M, and three actin-binding proteins—Wiskott-Alderich syndrome protein (WASP), WASP family member 2 (WASF2), and WASP-interacting protein 1 (WIPF1) (Fig. 1A).

A “RILK” domain at the C terminus of NiV-M is highly conserved with the actin-binding motif in SeV and hPIV-1 M (Fig. 1A). The RILK domain is located at the C terminus (orange) of NiV-M, protrudes outward of the NiV-M dimer, and is spatially away from the key motifs for membrane association (cyan) and dimerization (yellow) (Fig. 1B). In this domain, isoleucine-349 (I349) is identical across all sequences, and lysine-351 (K351) and arginine-348 (R348) are conserved among viral proteins (Fig. 1A). Additionally, both R348 and I349 are located on a β strand, while K351 is on a flexible region (Fig. 1B). We mutated R348 to lysine (K), and I349 and K351 to alanines (A) and fused them to an N-terminal 3xFLAG tag (fig. S1A). D344, located outside of the putative actin-binding domain, was mutated to asparagine (N). When expressed in 293T cells, both I349A and K351A pulled down less β-actin than the wild-type (WT) protein, with I349A even less than K351A (Fig. 1C). R348K and D344N did not show a significant difference in β-actin pulldown compared to that of WT (Fig. 1C). This suggests that both I349 and K351 residues are important in maintaining the interaction between NiV-M and β-actin. To analyze the role of NiV-M-actin interaction in VLP production, WT and mutant M were ectopically expressed in 293T cells, and the cell lysates and VLPs in the supernatant were analyzed by Western blot analysis. The normalized budding index indicates that the I349A and K351A mutants are less efficient in producing VLPs compared to the WT, whereas the D344N and R348K mutants produce VLPs at levels comparable to the WT (Fig. 1D). These findings indicate that the putative actin-binding domain at the C terminus of NiV-M, particularly residues I349 and K351, is critical for β-actin interaction and that this interaction is closely linked to efficient VLP production. I349A and K351A were selected for further analysis because they showed reduced actin-interaction and decreased VLP production.

Next, COS-7 cells were used to examine membrane protrusions induced by NiV-M constructs because these cells have fewer endogenous filopodia than PK13 and 293T cells and are therefore suitable for microscopic observations (Fig. 1E) (*10*). COS-7 cells stably expressing I349A exhibited fewer membrane protrusions compared to cells expressing the WT protein, while no significant difference was observed between K351A- and WT-expressing cells (Fig. 1E). These data indicate that a strong interaction between NiV-M and F-actin is required for generating membrane protrusions during VLP budding. Additionally, we analyzed the incorporation of β-actin into individual VLPs using confocal microscopy. NiV-VLPs were visualized through green fluorescent protein (GFP) fluorescence from GFP-tagged NiV-M constructs (green), while β-actin was labeled using an anti–β-actin antibody (magenta) (Fig. 1F). We detected a higher percentage of β-actin–positive VLPs (GFP^+^/β-actin^+^) in the supernatant collected from 293T cells expressing NiV-M-WT compared to those expressing I349A and K351A, indicating that actin incorporation into VLPs is impaired by these mutations (Fig. 1F). We confirmed that the GFP-tagged and FLAG-tagged NiV-M constructs (fig. S1A) showed a similar trend in VLP production (fig. S1, B and C). Collectively, these data suggest that altered actin interaction of NiV-M impairs membrane protrusion generation and actin incorporation into VLPs, highlighting a mechanistic link between actin interaction and viral assembly and budding.

### The nano-organization of NiV-M assembly sites is disrupted by F-actin depolymerization

**Fig. 2.**
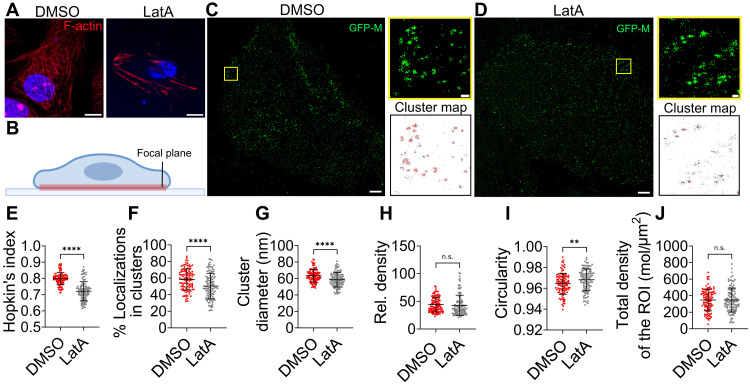
F-actin depolymerization alters the nano-organization of NiV-M. (**A**) PK13 cells were treated with 1 μM DMSO or 1 μM LatA for 5 min and stained with phalloidin Alexa Fluor 647. Images were taken using laser scanning confocal microscopy. Representative images of 10 to 20 cells collected from three independent experiments are shown. Scale bars, 1 μm. (**B**) The imaging plane is at the ventral membrane of PK13 on the cell–cover glass interface. (**C** and **D**) *x*-*y* cross section (600 nm thick in *z*) of the SMLM images of GFP-NiV-M at the ventral membrane of PK13 cells treated by DMSO (C) or LatA (D). The boxed regions are enlarged, and the cluster maps of the NiV-M localizations are shown. NiV-M clusters are masked in red, while the unclustered NiV-M localizations are gray. Scale bars, 1 μm and 200 nm. (**E**) The Hopkin’s index of NiV-M localizations in DMSO and LatA-treated cells. The percentage of localizations in clusters (**F**), cluster diameter (**G**), relative density (**H**), circularity (**I**), and total density of the region of interest (ROI) (**J**) are shown in dot plots. Sample size *n* = 106 (DMSO) and 115 (LatA) from 9 to 15 cells per group. Bars represent means ± SD. *P* value was obtained using Student’s *t* test with Welch correction. n.s., *P* > 0.05; **P ≤ 0.01; ****P ≤ 0.0001. Results from ≥3 independent experiments are shown.

We noticed that NiV-M copartitioned with vimentin to the cytoskeleton portion of the cell lysate, suggesting that NiV-M is mainly associated with F-actin (fig. S3A). PK13 cells treated with an F-actin depolymerization drug, latrunculin A (LatA), were smaller in cell size and showed fewer fine actin structures compared to the vehicle [dimethyl sulfoxide (DMSO)] control (Fig. 2A). We then examined the role of F-actin in NiV-M assembly by analyzing the nano-organization of NiV-M assembly sites using single-molecule localization microscopy (SMLM). The localization precision of our custom-built SMLM system is ~10 nm in the lateral direction and ~25 nm in the axial direction (fig. S2A) (*26*, *27*). It enables direct visualization and quantitative analysis of NiV-M nanoclusters and fine F-actin structures that are beyond the resolution limits of conventional diffraction-limited microscopy (fig. S2, B and C). GFP-tagged NiV-M constructs were used to identify NiV-M puncta under wide-field microscopy. SMLM images were acquired using Cy3B or Alexa Fluor 647 attached to the GFP tag. We verified that the labeling tags at the N terminus of NiV-M minimally affected NiV-M clustering (fig. S3, B to G). Images show that NiV-M forms nanoclusters at the ventral membrane of PK13 cells, consistent with those observed at the dorsal membrane of these cells (Fig. 2, B and C) (*27*). By visual inspection, NiV-M localizations form distinctive clusters in DMSO-treated PK13 cells (control) and less defined clusters in LatA-treated cells (Fig. 2, C and D). To analyze the partition of NiV-M localizations in clusters, NiV-M clusters were identified using a DBSCAN (density-based spatial clustering of applications with noise) algorithm that links closely situated localizations in a propagative manner (*26*, *28*), and the clusters were masked in red in cluster maps (Fig. 2, C and D). Smaller clusters and more unclustered localizations (gray) were observed for NiV-M localizations in LatA-treated cells compared to those in the control (Fig. 2, C and D, cluster map). We performed Hopkins’ analysis to evaluate the clustering tendency of NiV-M localizations. The Hopkins’ index indicates the extent of clustering, ranging from 0 (random distribution) to 1 (extreme clustering). The result shows that NiV-M localizations in LatA-treated cells are less likely to cluster compared to those in the control, demonstrated by a significantly lower Hopkin’s index (Fig. 2E). Consistently, we noticed that a lower percentage of NiV-M localizations segregated into clusters in LatA-treated cells (Fig. 2F). Additionally, NiV-M clusters were smaller upon LatA treatment (Fig. 2G), although they show similar density (Fig. 2H). We noticed that the NiV-M clusters in LatA-treated cells were less circular (Fig. 2I), consistent with the idea that NiV particles exhibit irregular shapes, as these NiV-M clusters likely serve as sites for virus assembly and budding (*27*, *29*). The total density of the selected regions was similar in both DMSO- and LatA-treated cells, indicating that these differences are not due to varying amounts of NiV-M localizations (Fig. 2J). Together, these results suggest that F-actin in host cells promotes NiV-M clustering at the PM, thereby enhancing NiV VLP production.

### The nano-organization of NiV-M assembly sites depends on its proximity to F-actin

**Fig. 3.**
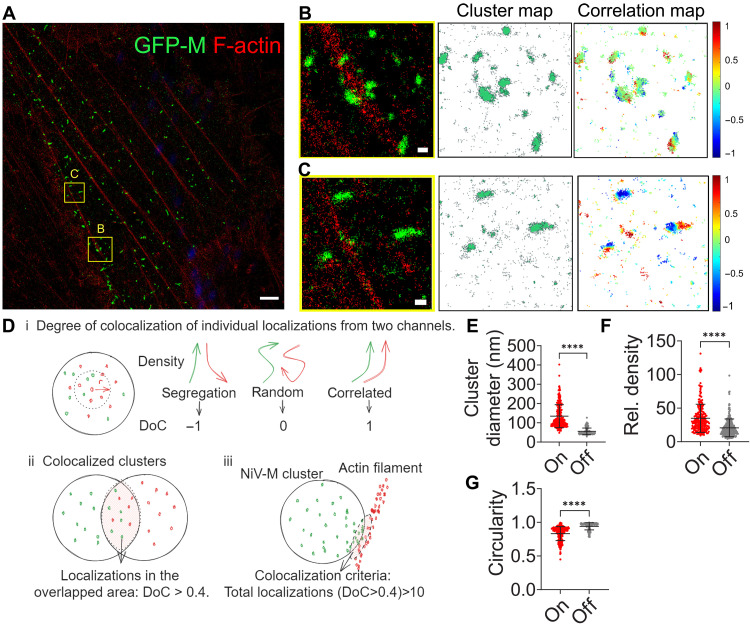
The nano-organization of NiV-M depends on its proximity to F-actin. (**A**) *x*-*y* cross section (600 nm in *z*) of GFP-NiV-M (green) and F-actin (red) at the ventral membrane of PK13 cells. (**B** and **C**) Left: The boxed regions in (A) are enlarged. Middle: The cluster map of NiV-M (green). Right: A heatmap of the degree of colocalization (DoC) value of NiV-M localizations, with −1 (segregation) in deep blue and 1 (correlation) in deep red. Scale bars, 1 μm in (A) and 200 nm in [(B) and (C)]. (**D**) (i) The density gradients of both channels are calculated along an increasing radius size (*r*) around each localization. The two density gradients are tested for correlation, resulting in a DoC value for each localization. [(ii) and (iii)] A NiV-M cluster was considered on the F-actin when the number of coclustered localizations (DoC ≥ 0.4) exceeded 10 (*26*, *40*). The diameter (**E**), relative density (**F**), and circularity (**G**) (the cluster is a true circle when circularity is 1) of NiV-M clusters on (*n* = 232) and off F-actin (*n* = 240) from 17 cells. Bars represent means ± SD. *P* value was obtained using Student’s *t* test with Welch’s correction. ****P ≤ 0.0001. Results from ≥3 independent experiments are shown.

To precisely analyze the role of F-actin in NiV-M assembly, we performed dual-color SMLM imaging of NiV-M and actin cytoskeleton on the ventral membrane of PK13 cells. The SMLM images show that NiV-M clusters are distributed around F-actin, with some clusters located close to F-actin and others far away (Fig. 3, A to C). To quantitatively compare NiV-M clusters with varying distributions relative to F-actin, we categorized them into two groups on the basis of their association with F-actin: NiV-M clusters that colocalized with F-actin (on) and those that did not (off). First, NiV-M clusters were identified using a DBSCAN algorithm (Fig. 3, B and C, cluster map). Next, the clusters were grouped on the basis of their coclustering with F-actin using a coordinate-based correlation assay for SMLM data (Fig. 3D) (*27*, *30*). The coclustering between NiV-M and F-actin was mainly observed at the border of the NiV-M clusters, marked by orange-red dots in the correlation map (Fig. 3, B and C). Our analysis shows that NiV-M clusters on F-actin are larger (Fig. 3E) and denser (Fig. 3F) than those off F-actin. NiV-M clusters associated with F-actin often displayed irregular shapes, whereas those distant from F-actin were predominantly round (Fig. 3G). These results show that the NiV-M nanoclustering is correlated with its proximity to F-actin.

### F-actin retains NiV-M assembly sites at the PM

**Fig. 4.**
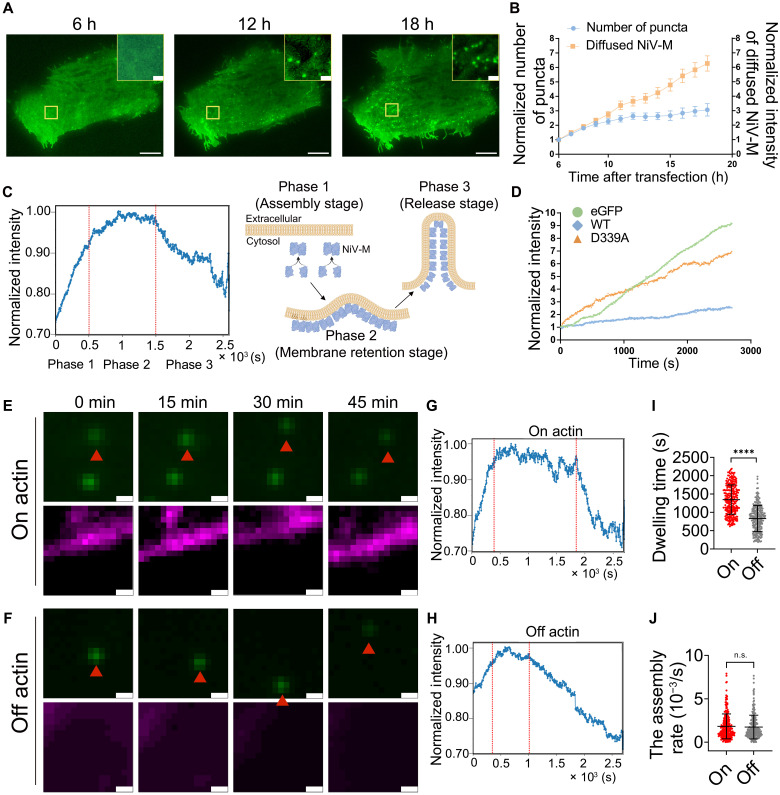
F-actin retains NiV-M assembly sites at the PM. (**A**) PK13 cells expressing GFP-NiV-M were monitored by TIRF microscopy at 6, 12, and 18 hours posttransfection. Scale bars, 10 and 1 μm. (**B**) PK13 cells expressing GFP-NiV-M were imaged at 6 to 18 hours posttransfection using epifluorescence illumination and TIRF. The number of puncta and the intensity of diffused NiV-M were normalized to that of 6 hours. h, hours. (**C**) The intensity profile of GFP-NiV-M was averaged from 250 tracks from 20 cells. Red dashed lines separate three phases. (**D**) The intensity of GFP signals in PK13 cells expressing monomeric eGFP, GFP-NiV-M, or GFP-NiV-M-D339A was monitored using epifluorescence illumination. (**E** and **F**) Representative images of PK13 cells expressing both GFP-NiV-M (green) and F-tractin-mCherry (magenta). Scale bars, 1 μm. (**G** and **H**) The intensity profiles of on-actin and off-actin GFP-NiV-M were averaged from 260 and 238 tracks from 30 to 40 cells, respectively. The dwelling time (**I**) and assembly rate (**J**) of on-actin and off-actin NiV-M tracks. Bars represent means ± SEM (B) and means ± SD [(I) and (J)]. *P* value was obtained using Student’s *t* test with Welch’s correction. n.s., *P* > 0.05; ****P ≤ 0.0001. Results from ≥3 independent experiments are shown.

We envision two possible mechanisms by which F-actin promotes NiV-M nanoclustering: (i) F-actin facilitates the recruitment of NiV-M molecules to the existing assembly sites, and (ii) F-actin stabilizes existing assembly sites and prevents their disassembly or premature release. To distinguish between these two scenarios, we determined the assembly dynamics of GFP-NiV-M by monitoring the intensity changes of individual punctum over time using total internal reflection fluorescence (TIRF) microscopy combined with single-particle tracking (SPT) analysis. NiV-M puncta started to appear on the PM at 6 hours posttransfection in PK13 cells and accumulated to an optimal level for microscopic observation and SPT analysis at 12 to 18 hours posttransfection (Fig. 4A). The signals were observed either as diffused distributions or as discrete puncta (Fig. 4A), which aligns with our previous SMLM observations that NiV-M nanoclusters were small and uniform in size (perceived as diffused signals under conventional fluorescence microscopy) within the cell interior, whereas larger clusters were detected at the cell periphery (*27*). We observed a consistent increase in the intensity of diffused NiV-M and a slower growth of the number of NiV-M puncta (Fig. 4B), suggesting that NiV-M molecules were first expressed in the diffused form and then assembled into puncta. We selected cells that harbored a low number of puncta at the beginning of observation and monitored the dynamics of their fluorescence intensity change at the ventral PM over a 45-min period at 5 s per frame using TIRF microscopy and SPT analysis. Because we observed membrane protrusions of ~1 μm in length induced by NiV-M in COS-7 cells (Fig. 1E), we did not analyze the intensity of the elongated puncta because they were likely virus budding sites that were fully assembled or at a late stage of assembly. Further, we noticed that the NiV-M nanoclusters were well spaced in SMLM (Fig. 3A) at the time of observation, and the puncta for intensity analysis were likely individual NiV-M assembly sites instead of large aggregations of NiV-M. Figure 4C is an averaged intensity profile of NiV-M puncta detectable over 75% of the total observation time. The intensity profile revealed three distinct phases: an initial rapid increase in fluorescence intensity (phase 1), followed by a stationary phase (phase 2) characterized by a stable intensity with minor fluctuations, and a final phase of fluorescence decay (phase 3, Fig. 4C). To distinguish the intensity changes associated with the behaviors of assembly sites from the overall intensity alteration within the cells, we imaged (i) NiV-M, (ii) an assembly-deficient mutant NiV-M-D339A (*10*), and (iii) monomeric enhanced GFP (eGFP) using epi-illumination to assess the overall intensity change. We observed a steady increase in the intensity of both diffused NiV-M WT and D339A, similar to the pattern seen with eGFP (Fig. 4D). By contrast, the intensity of NiV-M puncta follows a distinct three-stage pattern (Fig. 4C). Additionally, the intensity of diffused D339A increased more rapidly than that of the WT, suggesting that the diffused D339A was less efficient in aggregating into puncta, thereby confirming its assembly deficiency (Fig. 4D). These data inform us that (i) NiV-M puncta were derived from the assembly of diffused NiV-M at the PM (Fig. 4, B and D), and (ii) the rapid growth of fluorescence intensity of NiV-M puncta (phase 1 in Fig. 4C) was not due to an intensity change of the GFP signal (Fig. 4D), although phase 3 might be a combination of NiV-M puncta moving out of the TIRF illumination area (endocytosis or release) and photobleaching. Therefore, the NiV-M punctum intensity profile reflects three stages in the assembly and budding process: (i) the assembly stage where diffused NiV-M molecules are recruited to the PM and assemble into puncta, (ii) the membrane-retention stage during which the NiV-M puncta remain on the PM with minimal changes in intensity, and (iii) the release stage where the NiV-M puncta are either endocytosed or released into the extracellular space (Fig. 4C).

By coexpressing Ftractin-mCherry, a probe for F-actin, and GFP-NiV-M in PK13 cells, we observed that some NiV-M puncta (“on actin”; Fig. 4E) stayed on or moved along F-actin, while some moved laterally away from F-actin (“off actin”; Fig. 4F). We classified the “on-actin” and “off-actin” tracks by assessing the percentage of events colocalizing with F-tractin on individual tracks. Ideally, a colocalization rate of 100% indicates that a NiV-M punctum remains associated with actin filaments for the entire duration of the analysis, while a rate of 0% suggests no colocalization at any point during the time window. However, tracks with extreme colocalization values (i.e., 0 or 100%) are limited in number, which may compromise the statistical robustness of the analysis. To balance stringency and sample size, we defined a NiV-M track as on actin when the colocalization rate exceeded 98% and off actin when it was below 2% (fig. S4). The intensity profiles of on-actin (Fig. 4G) and off-actin (Fig. 4H) tracks were averaged from 200 to 300 tracks of each case. For the on-actin tracks, NiV-M puncta underwent distinctive assembly, membrane-retention, and release stages (Fig. 4G). However, the membrane-retention stage was short and not clearly distinguished from the release stage for the off-actin tracks (Fig. 4H). Our analysis of the duration of the membrane retention stage, measured by membrane dwelling time, shows that the on-actin tracks have a significantly longer membrane dwelling time than the off-actin tracks (Fig. 4I). The assembly rates, represented by the slope of the curve at the assembly stage, were similar between the on-actin and off-actin NiV-M tracks (Fig. 4J). These data indicate that F-actin stabilizes NiV-M assembly sites by preserving their association with the PM, rather than promoting the recruitment of additional NiV-M molecules to the assembly sites.

### The impaired F-actin interaction of NiV-M-I349A results in dispersed nanoclusters and reduced membrane retention time

**Fig. 5.**
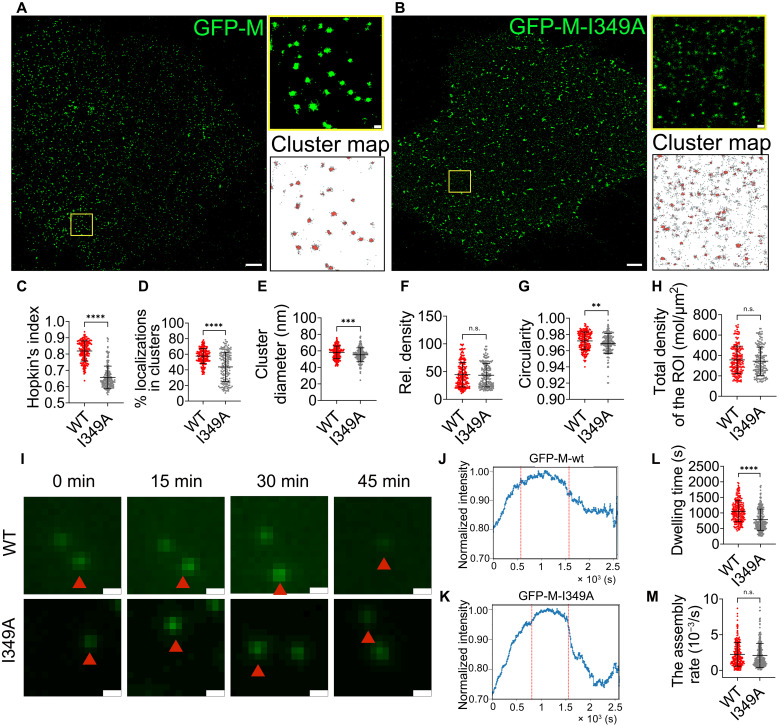
I349A alters the nano-organization of NiV-M and assembly dynamics. (**A** and **B**) *x*-*y* cross section (600 nm in *z*) of the SMLM images of GFP-tagged NiV-M-WT (A) and I349A (B) at the ventral membrane of PK13 cells. The boxed regions are enlarged, and the cluster maps of the NiV-M localizations are shown. Scale bars, 1 μm and 200 nm. (**C**) The Hopkin’s index of the localizations of NiV-M-WT and I349A. (**D** to **H**) The percentage of localizations in clusters (D), cluster diameter (E), relative density (F), circularity (G), and total density of the ROI (H). Sample size *n* = 186 (WT) and 164 (I349A) from 12 to 18 cells per group. (**I**) Representative images of PK13 cells expressing GFP-tagged NiV-M-WT and I349A. Scale bars, 1 μm. (**J** and **K**) The intensity profiles of NiV-M-WT and I349A puncta were averaged from 232 and 254 tracks from 30 to 40 cells, respectively. Red dashed lines separate three phases. The dwelling time (**L**) and assembly rate (**M**) of NiV-M-WT and I349A tracks. Bars represent means ± SD. *P* value was obtained using Student’s *t* test with Welch correction. n.s., *P* > 0.05; **P ≤ 0.01; ***P ≤ 0.001; ****P ≤ 0.0001. Results from ≥3 independent experiments are shown.

To further investigate whether direct interaction with actin plays a role in NiV-M assembly, we tested the nano-organization of actin-binding-deficient NiV-M mutants on cell membranes. Single-color SMLM images show that I349A forms more dispersed and smaller clusters than that of the WT (Fig. 5, A and B). We also observed a higher proportion of nonclustered I349A localizations compared to that of WT (Fig. 5, A and B, cluster map), consistent with the analyses showing a lower clustering tendency of I349A (Fig. 5C) and a lower percentage of the I349A localizations segregated into clusters (Fig. 5D). Similarly, clusters formed by I349A were smaller in size (Fig. 5E) but exhibited similar relative density (Fig. 5F) and more regular shapes compared to the WT (Fig. 5G), although the total density was comparable to that of WT (Fig. 5H). These results suggest that disrupting NiV-M-actin interaction leads to a more dispersed distribution of NiV-M on the PM, indicating either a deficiency in the assembly process or a breakdown of the existing assembly sites. We also analyzed the intensity change of the puncta formed by I349A using TIRF and SPT and compared it to that of WT as described above (Fig. 5, I to K). While the assembly rate of I349A was comparable to that of WT (Fig. 5M), its membrane dwelling time was markedly reduced (Fig. 5L). The nanoclustering and dwelling time of NiV-M at the PM likely reflect two distinct forms of its membrane association. The I349 residue, critical for NiV-M’s interaction with actin, appears to maintain both clustering and membrane dwelling by retaining NiV-M at the PM. This further supports the idea that the integrity of NiV-M assembly sites at the PM depends on F-actin. Consistently, K351A did not significantly alter the nano-organization or distribution of NiV-M (fig. S5, A to H) but led to a slightly longer dwelling time than that of WT at the PM (fig. S5, I to M). Because K351A has a stronger actin-binding affinity than I349A, these results suggest that the membrane association of NiV-M may be linked to its affinity for actin. Additionally, K351A induces a comparable level of membrane protrusions to that of WT (Fig. 1E). We speculate that K351A may affect the fission process, which is downstream of membrane association and protrusion formation.

### Arp2/3-driven F-actin branching retains NiV-M assembly sites on the membrane and promotes VLP budding and release

**Fig. 6.**
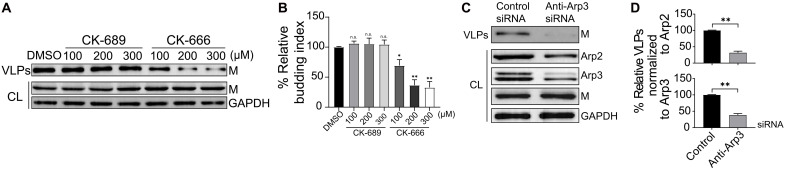
The Arp2/3 complex promotes NiV VLP production. (**A**) HeLa cells stably expressing 3xFLAG-NiV-M were treated with CK-689 and CK-666. Cell lysates and VLPs were collected and analyzed by Western blot. NiV-M was detected using a mouse anti-FLAG antibody. (**B**) Relative budding index is determined on the basis of integrated immunoblot density in (A). *P* value was obtained using one-way ANOVA with post hoc correction. n.s., *P* > 0.05; *P ≤ 0.05; **P ≤ 0.01. (**C**) HeLa cells stably expressing 3xFLAG-NiV-M were treated with control and anti-Arp3 siRNA. Cell lysates and VLPs were collected and analyzed by Western blot. NiV-M, Arp2, and Apr3 were detected using a mouse anti-FLAG antibody, a rabbit anti-Arp2, and a rabbit anti-Arp3 antibody. (**D**) VLP production was normalized to Arp2 and Arp3 expression levels based on the integrated immunoblot density in (C). Bars represent means ± SEM. *P* values were obtained using Student’s *t* test with Welch correction. n.s., *P* > 0.05; *P ≤ 0.05; **P ≤ 0.01. Results from ≥3 independent experiments are shown.

**Fig. 7.**
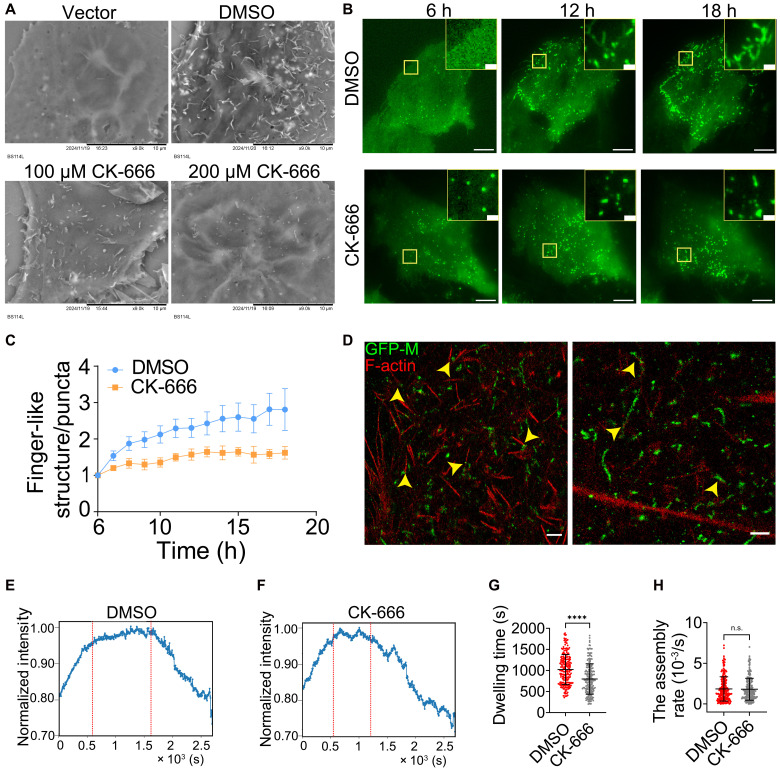
Arp2/3-driven F-actin branching retains NiV-M assembly sites on the membrane and promotes VLP budding. (**A**) COS-7 cells stably expressing 3xFLAG-NiV-M treated with DMSO and CK-666 were imaged by SEM. Scale bar, 10 μm. (**B**) PK13 cells expressing GFP-NiV-M were treated with DMSO and 200 μM CK-666 and monitored by TIRF microscopy. Scale bars, 10 and 1 μm. (**C**) The ratio of the number of finger-like structures to puncta was monitored in PK13 cells treated with DMSO or 200 μM CK-666. h, hours. (**D**) *x*-*y* cross section (600 nm in *z*) of an SMLM image of GFP-NiV-M (green) and F-actin (red) in PK13 cells. Arrowheads point to NiV-M at the end of F-actin (left) or along F-actin (right). Scale bars, 1 μm. (**E** and **F**) PK13 cells expressing GFP-NiV-M were treated by 200 μM DMSO or CK-666 and monitored by TIRF microscopy. The intensity profiles of NiV-M in DMSO- and CK-666–treated PK13 cells were averaged from 287 and 277 tracks from 30 to 40 cells, respectively. The dwelling time (**G**) and assembly rate (**H**) of NiV-M tracks on the PM in DMSO- and CK-666–treated cells. Bars represent means ± SEM (C) or means ± SD [(G) and (H)]. *P* values were obtained using Student’s *t* test with Welch correction. n.s., *P* > 0.05; ****P ≤ 0.0001. Results from ≥3 independent experiments are shown.

Our findings indicated that F-actin primarily stabilizes NiV-M assembly sites at the PM after assembly is complete, rather than facilitating the recruitment of additional NiV-M molecules to these sites. Because the Arp2/3 complex is known to nucleate F-actin branching and is important for both virus entry and escape (*14*), we hypothesized that Arp2/3-driven F-actin branching and polymerization are responsible for the membrane retention of NiV-M assembly sites. VLP production decreased significantly in HeLa cells treated with CK-666 (Fig. 6, A and B) while remaining at similar levels in cells treated with a CK-666 inactive analog, CK-689 (Fig. 6, A and B). Furthermore, an anti-Arp3 small interfering RNA (siRNA)–mediated knockdown of the endogenous Arp2 and Arp3 decreased VLP production in HeLa cells stably expressing NiV-M (Fig. 6, C and D). We did not observe a significant difference in the extent of VLP reduction when normalized to Arp2 and Arp3 expression levels (Fig. 6D). These results suggest that the Arp2/3 complex plays a role in VLP production. Next, we observed that the number of membrane protrusions in COS-7 cells stably expressing NiV-M was inversely correlated with the treatment dosage of an Arp2/3-inhibitor, CK-666 (Fig. 7A). We also observed many finger-like structures formed by NiV-M by TIRF at 6 to 18 hours posttransfection (Fig. 7B). These finger-like structures closely resembled the membrane protrusions seen in SEM images (Fig. 7A). The ratio of the number of finger-like structures to that of puncta increased over time in the control group (DMSO), suggesting that the finger-like structures likely developed through the transition of puncta (Fig. 7C). By contrast, we did not observe an increase of the finger-like structures over puncta in cells treated by CK-666 (Fig. 7C). At 200 μM, CK-666 disrupts the F-actin network in COS-7 cells, as evidenced by a loss of lamellipodia and a reduction of F-actin density (fig. S6, A and C). We noticed that NiV-M is mostly distributed at one end of F-actin (Fig. 7D, top) and along the F-actin (Fig. 7D, bottom) in the membrane protrusions, implying that NiV-M may be involved in the nucleation and polymerization of F-actin during membrane protrusion formation. These observations suggest that NiV-M hijacks the Arp2/3-mediated actin branching for membrane protrusion generation. To investigate whether the Arp2/3 complex is involved in the membrane retention of NiV-M assembly sites, we analyzed the NiV-M intensity profile using TIRF and SPT in PK13 cells treated with 200 μM CK-666. This treatment resulted in a reduction of F-actin density (fig. S6, B and D), decreased VLP production (fig. S6, E and F), and without substantially affecting the viability of PK13 cells (fig. S6G). A profound reduction in the membrane-dwelling time of NiV-M puncta was observed in cells treated with CK-666 compared to that in control-treated cells (Fig. 7, E to G), suggesting that the Arp2/3-driven actin branching is critical for membrane retention of NiV-M puncta. Additionally, it is unlikely that Arp2/3 is substantially involved in the assembly stage because the assembly rate was not affected by CK-666 treatment (Fig. 7, E, F, and H). Together, these results highlight the role of the Arp2/3-driven actin polymerization and branching in promoting NiV VLP production. This process is facilitated by F-actin–dependent membrane retention, which may depend on the interaction between NiV-M and F-actin.

## DISCUSSION

Assembly and budding are tightly coupled processes, particularly for viruses like paramyxoviruses that assemble at the PM (*31*). Biochemical and proteomic studies have revealed cytoskeletal remodeling during paramyxovirus infection and identified interactions between viral proteins and actin or actin-binding proteins (*18*, *24*, *32*, *33*). However, dissecting the specific role of the actin cytoskeleton in virus assembly and budding remains challenging. Traditional biochemical assays rely on virus release as a bulk readout, which overlooks the finer details and dynamic steps involved in the assembly and budding processes. Conventional fluorescence microscopy lacks the spatial resolution to resolve and quantitatively determine the nanoscale organization of viral proteins at the PM, while electron microscopy, although offering high resolution, is constrained by its inability to capture dynamic processes in living cells. Super-resolution microscopy bridges this gap by enabling the visualization and analysis of virus-induced nanoscale structure and dynamic interactions in situ, offering insights into the spatial and temporal coordination of viral assembly and budding. In this study, we use TIRF microscopy to track intensity change of individual assembly sites in real time, which leads to the distinction of assembly, membrane retention, and release stages (Fig. 4C); meanwhile, we use SMLM to quantitatively analyze the spatial distribution and organization of NiV-M on a nanoscale, which informs the structural integrity of individual assembly sites (Figs. 2 and 3).

Combined temporal and spatial analyses of individual NiV-M assembly sites following the disruption of actin dynamics and NiV-M-actin interaction reveal that the F-actin dependent integrity of NiV-M assembly sites is associated with sustained membrane retention, but not with changes in the assembly rate. Together with the results showing that Arp2/3 promoted VLP production by facilitating the generation of NiV-M–induced membrane protrusions, our data demonstrate that (i) NiV-M assembly sites remain on the PM after assembly completion; (ii) F-actin is important in this retention by preventing NiV-M disassembly or dissociation from the PM; (iii) membrane retention of NiV-M assembly sites is likely linked to Arp2/3-driven F-actin polymerization and branching, which further facilitates membrane protrusion generation. We propose a model by which NiV-M couples F-actin for NiV assembly and budding: Following dimerization and binding to PS and PI(4,5)P_2_ on the PM, NiV-M interacts with F-actin to maintain the nanoscale organization and stable association of the NiV-M assembly sites at the PM, ensuring the proper formation of the viral assembly sites. Meanwhile, NiV-M co-opts with the Arp2/3-driven F-actin branching to generate membrane protrusion, supporting the membrane curvature required for virus budding. Our findings suggest a dual role of F-actin in NiV assembly and budding: (i) F-actin acts as a scaffold that maintains spatial organization and membrane association of the NiV-M assembly sites at the PM, and (ii) F-actin promotes NiV-M–induced membrane protrusion and VLP budding.

Existing evidence suggests a positive role of actin in paramyxovirus assembly and budding. The involvement of actin in paramyxovirus assembly and budding has been demonstrated by the presence of actin filaments in virus-induced structures and mature virions, as well as through biochemistry and proteomics analysis of interactions between viral proteins and actin or actin-binding proteins (*17*, *34*). Actin remodeling was observed in SeV-infected madin-darby canine kidney (MDCK) cells, in which actin bundles at the cell periphery disappeared and both β- and γ-actins formed puncta on the apical surface of the infected cells at a later infection stage (*18*). We show membrane protrusions in NiV-M–expressing COS-7 cells (Fig. 1E), and these membrane protrusions are less prominent when the NiV-M-actin interaction is disrupted by the I349A mutant or the actin branching in COS-7 cells is inhibited by CK-666 (Fig. 7A). Additionally, MeV-specific buds and virion release from infected cells were significantly reduced upon the disruption of actin microfilaments by cytochalasin B and D (*35*). Suppression of β- and γ-actins leads to an 80% decrease in virus production (*18*). Moreover, actin remodeling and virus production are thought to be driven by SeV-M, as evidenced by two findings: (i) a SeV mutant carrying an altered M protein fails to induce actin remodeling or produce virus particles (*18*) and (ii) a mutation in a putative actin-binding motif at the C terminus of SeV-M significantly reduces virion release (*25*). Similarly, our data suggest that actin contributes to NiV assembly and budding. For example, the disruption of actin polymerization by LatA leads to breakdown of the NiV-M assembly sites (Fig. 2). Inhibition of F-actin branching by CK-666 or Arp3 knockdown leads to reduced membrane retention of assembly sites (Fig. 7, E to G), fewer membrane protrusions (Fig. 7, A to C), and decreased VLP production (Fig. 6).

Studies on viruses of different families reveal opposing effects of actin cytoskeleton on virus assembly and budding. A recent study that combined SMLM and functional analyses shows that HIV Gag prefers to assemble at areas with low F-actin density at the PM, and decreased actin branching favors HIV Gag assembly and virus production in HIV-infected T cells (*23*). Another study also shows that the F-actin levels are reduced in Gag-positive regions of the PM, compared to adjacent Gag-negative regions in HeLa cells infected with HIV-1 strain NL4-3 pseudotyped with VSV-G (*36*). Both studies show that cellular factors capable of actin depolymerization (MICAL-I) or debranching (Arpin) promote HIV-1 virus release (*23*, *36*). In contrast, our SMLM data show that a close proximity to or direct interaction with F-actin enhances NiV-M clustering, resulting in larger and more irregularly shaped clusters resembling viral assembly sites (Figs. 3 and 5). We also noticed that NiV-M clusters are mostly at the end or distributed along F-actin (Fig. 7D), implying that NiV-M may be responsible for nucleating and polymerization of F-actin. Inhibiting actin branching by CK-666 and siRNA targeting Arp2/3 complex results in a decrease in NiV VLP production (Fig. 6). Although these opposing results may be due to different virus types, cell types, and experimental systems, they may also suggest that the F-actin network may act as a barrier to virus release or be actively recruited by viral proteins to facilitate virus assembly and budding in membrane retention and protrusion generation.

The function of actin in NiV assembly and budding is conveyed via its interaction with NiV-M.

A putative actin-binding motif is mapped to the C terminus of NiV-M by protein sequence alignment (Fig. 1, A and B). This motif spans a β strand and a flexible tail, with I349 in the β strand and K351 in the flexible tail. Both residues are exposed on the protein surface (Fig. 1B). Mutating either residue reduces acting binding (Fig. 1C) and VLP production (Fig. 1D). Because I349A has a weaker interaction with actin than K351A (Fig. 1C), it demonstrates a stronger impact than K351A on NiV-M–induced membrane protrusion formation, clustering, and membrane retention. NiV-M clustering and membrane dwelling time are distinct manifestations of its membrane association, detected by different methods. The membrane association of NiV-M is further linked to NiV-M’s actin-binding ability (Figs. 4 and 5), F-actin integrity (Figs. 2 and 3), and Arp2/3-driven actin branching and polymerization (Fig. 7, E to G). Our data indicate that membrane protrusion formation depends on NiV-M’s direct interaction with actin (Fig. 1E) and Arp2/3-mediated F-actin branching and polymerization (Fig. 7, A to C). Collectively, our results suggest that the strength of NiV-M-actin interaction modulates key steps in virus budding, including membrane curvature generation, stabilization of assembly sites, and efficient particle release.

In conclusion, our findings reveal that NiV-M co-opts with F-actin to stabilize NiV-M assembly sites at the PM and promote membrane curvature for VLP release. These findings provide mechanistic insight into how NiV-M exploits the actin cytoskeleton to coordinate spatial organization and membrane remodeling during viral assembly and budding, processes that have been challenging to resolve using conventional approaches. However, given that the role of F-actin in viral assembly and budding can vary depending on cell type, virus strain, and experimental system (*37*), further validation in more biologically relevant systems, such as noninfectious replicons or surrogate viruses, is needed. This is especially important considering the biosafety level 4 (BSL-4) restrictions associated with studying live NiV. Future studies will also be required to determine whether these mechanisms are conserved across other paramyxoviruses or related enveloped viruses.

## MATERIALS AND METHODS

### Plasmids

The plasmids expressing monomeric eGFP- and 3xFLAG-tagged NiV-M were adapted and modified from a previous study (*38*). NiV-M, NiV-M-D344N, R348K, I349A, and NiV-M-K351A were produced by site-directed mutagenesis using pcDNA 3 plasmids coding for GFP- and/or FLAG-tagged NiV-M. The plasmid expressing pLV-Ftractin-mCherry (Addgene, plasmid no. 85131) is a gift from T. Meyer. The Ftractin-mCherry gene was ligated into a pcDNA3 expression plasmid flanked by Kpn I and Eco RI restriction sites. For stable cell line establishment, cDNA fragments coding for FLAG-tagged NiV-M, NiV-M-I349A, and FLAG-NiV-M-K351A gene were ligated into a pQCXIP expression plasmid flanked by Pac I and Eco RI restriction sites, respectively. MLV-Gag/Pol was a gift from C. Liang at McGill University. The VSV-G envelope expressing plasmid, pmd2.G, was a gift from D. Trono (Addgene, plasmid no. 12259).

### Cell lines and tissue culture

PK13 [American Type Culture Collection (ATCC), CRL-6489; pig kidney epithelial cells], COS-7 (ATCC, CRL-1651; Grivet kidney fibroblast cells), human embryonic kidney (HEK) 293T (ATCC, CRL-3216; human embryonic kidney epithelial cells), and HeLa (ATCC, CCL-2; human cervix adenocarcinoma epithelial cells) cells were cultured at 37°C and 5% CO_2_ in Dulbecco’s modified Eagle’s medium (Sigma-Aldrich, D6429) complemented with 10% fetal bovine serum (Invitrogen, 12483-020). Cells were passaged using phosphate-buffered saline (PBS; Invitrogen, 10010-049) and 0.25% trypsin-EDTA solution (Invitrogen, 25002-072). Cells were monitored routinely for mycoplasma contamination using a mycoplasma detection PCR kit (Applied Biological Materials, G238). To generate stable cell lines, retroviral pQCXIP vectors encoding FLAG-tagged NiV-M constructs (4 μg), MLV-Gag/Pol (4 μg), and pmd2.G (0.5 μg) plasmids were cotransfected using polyethylenimine (PEI) at 1 mg/ml (Polysciences, 23966-100) to HEK293T cells cultured in a 10-cm dish. Viral supernatant was collected 72 hours posttransfection to infect COS-7 or HeLa cells. After 3 days of infection, puromycin selection was applied for 21 days to establish a stable cell line expressing the FLAG-tagged NiV-M constructs.

### Drugs, siRNA, and antibodies

Drugs used in this study are: LatA (Calbiochem, 428021), CK-666 (Calbiochem, 182515), CK-689 (Calbiochem, 182517), and DMSO (Sigma-Aldrich, D8418-100ML). Antibodies used in this study are as follows: anti-FLAG mouse monoclonal antibody (Sigma-Aldrich, F1804); anti-GFP goat antibody (Abcam, ab5450); anti-GFP rabbit antibody (Invitrogen, G10362); anti–β-actin mouse antibody (Sigma-Aldrich, A2228); anti–glyceraldehyde-3-phosphate dehydrogenase (GAPDH) mouse antibody (Sigma-Aldrich, CB1001); anti-Arp3 rabbit antibody (Abcam, ab181164); anti-Arp2 rabbit antibody (Abcam, ab129018); anti-vimentin rabbit antibody (Abcam, ab92547); anti-goat donkey polyclonal antibody, Alexa Fluor 647 conjugated (Invitrogen, A21447); anti-rabbit donkey polyclonal antibody, Alexa Fluor 647 conjugated (Invitrogen, A16025); phalloidin Alexa Fluor 647 (A22287); anti-goat donkey polyclonal antibody, horseradish peroxidase (HRP) conjugated (Jackson ImmunoResearch, 705-035-147); anti-mouse goat polyclonal antibody, HRP conjugated (Bio-Rad, 1705047); and anti-rabbit goat polyclonal antibody, HRP conjugated (Bio-Rad, 1706515). The cy3B (Cytiva, PA63101) is conjugated to the donkey anti-goat antibody (Jackson ImmunoResearch, 705-005-003) and donkey anti-rabbit antibody (Jackson ImmunoResearch, 711-005-152) in-house by Ablabs. The siRNAs used in this study are siGENOME SMARTpool human ACTR3 (M-012077-01-0005) and nontargeting siRNA pool (D-001206-13-05).

### VLP production and purification

To determine the VLP production of various NiV-M constructs, HEK293T cells were transfected by FLAG-tagged or GFP-tagged NiV-M constructs and pcDNA3 vector at a 1:2 ratio by using PEI at 1 mg/ml. At 48 hours posttransfection, the supernatant of the cell culture was collected for VLP purification. To study the effect of Arp2/3 on NiV VLP production, PK13 or HeLa cells stably expressing FLAG-tagged NiV-M were seeded in six-well plates and treated with DMSO; 100, 200, and 300 μM CK-666; and CK-689 at 6 hours after seeding. After 48 hours of drug treatment, the supernatant was collected for VLP purification. Alternatively, HeLa cells stably expressing FLAG-tagged NiV-M were transfected by 20 nM anti Arp2/3 siRNA using 3 μl of RNAiMAX (Invitrogen, 13778075). At 48 hours posttransfection, the supernatant of the cell culture was collected. For VLP purification, the supernatant was filtered using 0.45-μm polyethersulfone (PES) membrane (VWR, 76479-020) and subjected to ultracentrifugation on a 20% sucrose cushion at 125, 392 relative centrifugal force (rcf) with an SW 41 rotor (Beckman) for 90 min. The VLP-containing pellets were resuspended in 5% sucrose-NaCl-Tris-EDTA (sucrose-NTE) buffer and stored in −80°C. The cells from each different treatment were lysed, and both cell lysates and purified VLPs were subjected to Western blot analysis to evaluate VLP production activity.

### Western blot analysis

The virus-producing cells were lysed using radioimmunoprecipitation assay buffer (Sigma-Aldrich, 20-188) supplemented with EDTA-free protease inhibitor cocktail (Sigma-Aldrich, 11836170001). The cell lysates were collected after centrifuge at 16,000*g* for 20 min at 4°C. VLP and cell lysate were supplemented with 1× SDS loading dye [60 mM tris-HCl (pH 6.8) (tris base; Millipore, 648311-1kg), 2% SDS (Sigma-Aldrich, L3771), 10% glycerol (Sigma-Aldrich, G5516-1 L), and 0.025% Brophenol blue (Sigma-Aldrich, 114391-5G)] and 15 mM dithiothreitol (DTT; Thermo Fisher, R0861) and heated at 95°C for denature. The denatured VLP and cell lysates were separated on a 10% polyacrylamide gel for SDS–polyacrylamide gel electrophoresis (PAGE). Proteins were then transferred to an activated polyvinylidene difluoride (PVDF) membrane (Millipore, IPVH00005), with pore size of 0.45 μm. The PVDF membrane was then blotted using 1% bovine serum albumin (BSA; Sigma-Aldrich, A9647) blocking buffer in PBS for 90 min at room temperature and incubated with primary antibodies. An HRP-conjugated goat anti-mouse, HRP-conjugated goat anti-rabbit, or donkey anti-goat secondary antibody was used for protein detection. The membranes were then developed with Bio-Rad Western ECL substrate (Bio-Rad, 170560). Membrane images were acquired using the ChemiDoc MP Imaging System (Bio-Rad). The relative budding index is determined as described previously (*10*). Specifically, the expression of NiV-M constructs and GAPDH control was determined by measuring the integrated intensity of the corresponding bands by densitometry in ImageJ. The band intensity of NiV-M constructs in the cell lysate was normalized to that of the GAPDH control, and, then, the normalized band intensity of NiV-M mutants was further normalized to that of WT. Similarly, the level of the NiV-M mutants in VLPs was normalized to that of the NiV-M-WT. The relative budding index was then determined by calculating the ratio of normalized NiV-M expression in VLP to that of cell lysate.

### Coimmunoprecipitation

HEK293T cells were transfected with the following plasmids: (i) 3xFLAG-NiV-M-WT, (ii) 3xFLAG-NiV-M-I349A, and (iii) 3xFLAG-NiV-M-K351A. At 48 hours posttransfection, cells from one well of a six-well plate were washed with PBS and lysed in 200 μl of lysis buffer provided with the μMACS DYKDDDDK isolation kit (Miltenyi Biotec, 130-101-591) and supplemented with protease inhibitors. Cells were isolated on ice for 30 min. Cell debris was removed by centrifuge at 16,000*g* for 20 min at 4°C. Cell lysate (60 μl) was set aside for immunoblot analysis, and the rest was used for immunoprecipitation, as recommended by the manufacturer. Anti-DYKDDDDK microbeads (6 μl; Miltenyi Biotec, 130-101-591) were added to 140 μl of cell lysates and incubated for 30 min on ice. μ Columns (Miltenyi Biotec, 130-042-701) were prepared according to the manufacturer’s instructions. Lysate was run over the columns, and microbeads were washed according to the manufacturer’s instructions. Preheated elution buffer (20 μl; 95°C) was added to the column before eluting the bound immunoprecipitated protein in 50 μl of elution buffer. Elute was separated by 10% SDS-PAGE, and proteins were immunoblotted by mouse anti-FLAG and mouse anti–β-actin antibodies. The HRP-conjugated goat anti-mouse and goat anti-rabbit secondary antibodies were used for protein detection. The integrated intensity of the NiV-M and β-actin band was measured by densitometry using ImageJ. The % actin pulldown is the ratio of β-actin over NiV-M in the immunoprecipitation (IP) group over that of the input group. The ratio of the NiV-M group is set as 100%.

### Immunofluorescence for confocal microscopy to visualize VLPs

To visualize protein composition in VLPs, VLPs expressing GFP-NiV-M were bound to coverslips (Marienfeld, no. 1.5H, 18 mm) coated by 2.5 μg of fibronectin at 37°C for 4 hours, followed by fixation using PBS containing 4% paraformaldehyde (PFA). VLPs were incubated with BlockAid blocking solution (Life Technologies, B10710) at room temperature for 1 hour and stained with mouse anti–β-actin and donkey anti-mouse antibody conjugated to Alexa Flour 647. The percentage of VLPs containing β-actin was determined as a ratio of the number of GFP^+^/β-actin^+^ particles over the number of GFP^+^ particles. Images were acquired using a laser scanning confocal microscopy Zeiss LSM710, and data analysis was performed using Imaris (Oxford Instruments).

### Immunofluorescence for SMLM

For SMLM imaging, 1 × 10^5^ PK13 cells were seeded on coverslips (Marienfeld, no. 1.5H, 18 mm) coated with 2.5 μg of fibronectin (Sigma-Aldrich, F4759-2mg) in a 12-well plate, and transfected with 0.3 μg of GFP- NiV-M constructs with 0.7 μg of pcDNA 3 plasmids using Lipofectamine 3000 (Invitrogen, L3000015) on the following day. In the case of drug treatment, PK13 cells expressing GFP-NiV-M were treated by using 1 μM DMSO or LatA for 5 min before fixation. The protocol for immunofluorescence (IF) of the actin cytoskeleton was adapted from Huang *et al.* (*39*).

For dual-color imaging of actin cytoskeleton and NiV-M constructs, cells were fixed at 20 to 24 hours posttransfection with 0.3% glutaraldehyde (Sigma-Aldrich, G5882-50ml) and 0.25% Triton X-100 (Sigma-Aldrich, T8787-50ML) in cytoskeleton buffer [CB; 10 mM MES (Sigma-Aldrich, 475893; pH 6.1), 150 mM NaCl (Sigma-Aldrich, S9888-1KG), 5 mM EGTA (Sigma-Aldrich, E3889-10G), 5 mM glucose (Sigma-Aldrich, G8270-1KG), and 5 mM MgCl_2_ (Sigma-Aldrich, M8266-100G)] for 1 to 2 min, followed by a second fixation step using 400 μl of 2% glutaraldehyde (Sigma-Aldrich, G5882-50ml) in CB for 10 min. Cells were treated with 1 ml of 0.1% NaBH_4_ (Sigma-Aldrich, 452882-5G) (freshly prepared in PBS) for 7 min to reduce background fluorescence. For IF of NiV-M, cells were fixed with PBS containing 4% PFA (Electron Microscopy Sciences, 50980487) and 0.2% glutaraldehyde for 90 min at room temperature followed by permeabilization using 0.1% Triton X-100 in PBS. After fixation and permeabilization, cells were incubated with signal enhancer image-IT-Fx (Life Technologies, I36933) for 30 min at room temperature and then blocked using BlockAid blocking solution for 1 hour at room temperature. The GFP-tagged NiV-M and mutants were detected by an anti-GFP rabbit (Invitrogen, G10362) or goat antibody (Abcam, ab5450) and a cy3B-conjugated donkey anti-rabbit or anti-goat secondary antibody. Cells were incubated with primary antibody overnight at 4°C and then with the secondary antibody for 1 hour at room temperature. Each antibody incubation was followed by five PBS washes, 5 min each time. The F-actin was stained using 0.5 μM Alexa Fluor 647–phalloidin (Invitrogen A22287), followed by three PBS washes. Cells were then fixed in PBS containing 4% PFA for 10 min at room temperature.

### SMLM setup, imaging, and data analysis

SMLM was performed on a custom-built microscopy described previously (*26*, *29*). Briefly, the microscopy was built upon an apochromatic TIRF oil-immersion objective lens [Nikon, 60×; numerical aperture (NA), 1.49]. Four lasers were used for excitation: a 639-nm laser (MRL-FN-639, 500 mW) for exciting Alexa Fluor 647, a 532-nm laser (MGL-III-532-300 mW) for exciting cy3B, a 488-nm laser (MBL-F-473-300 mW) for exciting GFP, and a 405-nm laser (MDL-III-405-100 mW) for reactivating Alexa Fluor 647 and cy3B. The emission fluorescence was separated using appropriate dichroic mirrors and filters (Semrock) and detected by electron-multiplying charge-coupled devices (Ixon, Andor). A feedback loop was used to control the sample drift to <1 nm laterally and 2.5 nm axially. Fluorescence beads (Life Technologies, F8799) were added to samples as fiducial markers for drift control. Samples were immersed in imaging buffer {Tris-NaCl (TN) buffer [50 mM tris (pH 8.0) and 10 mM NaCl], glucose oxidase (0.5 mg/ml; Sigma-Aldrich, G2133-50KU), catalase (40 μg/ml; Sigma-Aldrich, C100), 10% glucose, and 50 mM mercaptoethylamine (Sigma-Aldrich, 641022) or 143 mM 2-mercaptoethanol (Sigma-Aldrich, 444203) for dual-color SMLM imaging}. For SMLM imaging, samples were exposed to a laser power density of 1 kW/cm^2^ for the 639- and 532-nm lasers to activate Alexa Fluor 647 and cy3B, respectively. A total of 40,000 images were acquired at 50 Hz to reconstruct one SMLM image. Custom-written software in MATLAB (MathWorks) was used to reconstruct SMLM images. Clusters of NiV-M localizations were identified and characterized using ClusDoC (*40*). The minute points and ε for DBSCAN were set at 4 and 20, respectively (*26*). The degree of colocalization (DoC) assay was performed using ClusDoc. A DoC value is generated for individual localizations. The colocalized clusters contain more than 10 localizations with a DoC value greater than 0.4 (*28*).

### IF for confocal microscopy to determine the F-actin density

To quantify F-actin density, COS-7 and PK13 cells expressing NiV-M were fixed, permeabilized, and stained with Alexa Fluor 647–conjugated phalloidin, following the protocol described in the “Immunofluorescence for SMLM” section. **Z**-stack images were acquired using a Zeiss LSM780 confocal microscope with a 350-nm step size. Total phalloidin fluorescence intensity was measured as previously described (*41*). For each condition, the total phalloidin intensity from ~10 cells was averaged and normalized to the control group to calculate the percentage of normalized F-actin density.

### Cytoskeleton extraction assay

We used a protocol described previously to extract the cytoskeleton and determine the association of NiV-M on the cytoskeleton (*42*). PK13 cells were transfected with FLAG-tagged-NiV-M for 48 hours and subsequently washed with PBS. The cells were then incubated with cellular extraction buffer [50 mM Pipes (Sigma-Aldrich, P1851-25G), 50 mM NaCl, 5% glycerol, 0.1% NP-40 (Sigma-Aldrich, NP40S-100ML), 0.1% Triton X-100, and 0.1% Tween 20 (Sigma-Aldrich, P9416-100ML)] and cytoskeleton wash buffer (50 mM tris-HCl, pH 7.5) to obtain the soluble compartment (S). For the nuclear compartment (N), cells were incubated with nuclear extraction buffer [Benzonase nuclease (10 U/ml; Sigma-Aldrich, E1014-5KU) and 10 mM MgCl_2_ and 2 mM CaCl_2_ (Sigma-Aldrich, 21115-1ML) in 50 mM tris-HCl buffer (pH 7.5)]. Next, cells were washed with cellular extraction buffer and incubated with cytoskeletal solubilization buffer (1% SDS) to isolate the cytoskeletal compartment (C). The association of 3xFLAG-NiV-M with N, C, and S compartments was determined by Western blot analysis. Vimentin served as a control for compartment C, and GAPDH served as a control for compartments S and N. The extracts were supplemented with 1× SDS loading dye and 15 mM DTT and heated at 95°C for denature. Then, the extracts were separated on a 10% polyacrylamide gel for SDS-PAGE. Proteins were then transferred to an activated PVDF, with pore size of 0.45 μm. The PVDF membrane was then blotted using 1% BSA blocking buffer in PBS for 90 min at room temperature and incubated with primary antibodies (mouse anti-FLAG antibody, rabbit anti-vimentin antibody, and mouse anti-GAPDH antibody). An HRP-conjugated goat anti-mouse or HRP-conjugated goat anti-rabbit was used for protein detection. The membranes were then developed with Bio-Rad Western ECL substrate. Membrane images were acquired using ChemiDoc MP Imaging System.

### Scanning electron microscopy

COS-7 cells stably expressing FLAG-tagged NiV-M WT or mutants were seeded on coverslips (Marienfeld, no. 1.5H, 12 mm) coated with 1.25 μg of fibronectin (Sigma-Aldrich, F4759-2mg) in a 24-well plate. After 24 hours, cells were fixed with 2.5% (w/v) glutaraldehyde in 0.1 M sodium cacodylate buffer (Sigma-Aldrich, 70114-10ML-F) for 30 to 60 min at room temperature, followed by three PBS washes, 10 min each time. Then cells were fixed in precooled 1% (v/v) osmium tetroxide (Sigma-Aldrich, 251755-5ML) for 30 min at room temperature, followed by three PBS washes. Cells were then dehydrated through a graded ethanol series (25, 50, 75, 95, and 100%) and dried using a Tousimis 931 critical point dryer (Leica EM CPD300). Sputter coating was conducted using a sputter coater (Leica EM ACE200). Images were captured at ×9000 magnification using scanning electron microscopy (Hitachi TM-1000).

### TIRF microscopy and SPT

For SPT experiments, PK13 cells transfected by GFP-tagged NiV-M constructs or GFP-NiV-M and Ftractin-mCherry were subjected to TIRF-M imaging at 12 to 18 hours posttransfection. In case of CK-666 treatment, 200 μM CK-666 was added to the growth medium of PK13 cells transfected by GFP-NiV-M at 6 hours posttransfection and subjected to TIRF imaging at 12 to 18 hours posttransfection. Imaging was performed by a Zeiss TIRF microscopy equipped with a 63× NA 1.46 objective lens at TIRF illumination. A live cell chamber (Tokai Hit) maintained a 37°C and 5% CO_2_ atmosphere. Images were acquired at 5 s per frame for 45 min. To analyze the fluorescence intensity change of individual NiV assembly sites, we used SPT analysis to track NiV-M puncta over time. The image analysis was performed in four steps by Imaris (Oxford Instruments): (i) spot detection with background correction, (ii) tracking, (iii) track filtering, and (iv) track statistics. The tracks that lasted >75% of the total time points were included in further analysis. The fluorescence intensity of the NiV-M puncta at individual time points in each track was extracted and plotted as a curve to reflect the dynamics of intensity change of a NiV-M assembly site. Several hundreds of fluorescence intensity curves obtained from NiV-M tracks in PK13 cells were used to identify a reference pattern of the NiV-M fluorescence dynamics according to their distance to the core in the *t*-SNE (*t*-distributed stochastic neighbor embedding) plot. The *t*-SNE plot was generated as previously described (*26*). This reference pattern was used to select biologically significant tracks in all treatment groups according to their fluorescence intensity curves. This selection was performed by calculating the correlation coefficient of the intensity curve of a track with the reference, and tracks with a correlation coefficient ranked in the top 20% were included in further analysis. The fluorescence curves of the selected tracks were averaged to obtain an overall pattern of the fluorescence dynamics of each treatment group. The phase identification and assembly rate determination were performed using custom-written Python codes available on Zenodo platform (https://doi.org/10.5281/zenodo.15860700). Briefly, the phase was identified by determining the inflection points of the intensity curve by using a polynomial fitting shown in Eq. 1

y=anxn+an−1xn−1+…+a1x+a0 (1)

where $a_{n}$ are coefficients, y is the fluorescence intensity of NiV-M puncta, x is the time points, and n is the degree of polynomial optimized to 3 in our dataset. The polynomial fitting curve exhibited a snake-like trend, characterized by an initial increase, followed by a plateau, and then a decrease. The plateau (phase 2) is the region between the two infection points, representing the membrane-dwelling stage. The inflection points that mark the shoulder of the curve were determined by calculating second derivatives. Phase 1 is the initial increase region ending at the first inflection point, representing the assembly stage. We determined the assembly rate by averaging the derivatives at the first, second, third, and fourth quartiles of phase 1. Phase 3 begins at the second inflection point and continues to the end, representing the virus release stage.

Similarly, the fluorescence intensity of diffused GFP-tagged NiV-M constructs was determined using the Zeiss TIRF microscopy at epi-illumination in live cell chamber. Images were acquired at a 1-hour interval over 18 hours or at 5-s interval over 45 min, beginning at 6 hours posttransfection of PK13 cells with GFP-NiV-M. To monitor the dynamic changes of membrane protrusions induced by NiV-M, PK13 cells transfected by GFP-NiV-M were treated by 200 μM CK-666 treatment at 6 hours posttransfection and subjected to TIRF-M immediately. Images were acquired at a 1-hour interval over 18 hours. Puncta and finger-like structures in each image were identified using Imaris. The ratio of the number of finger-like structures to that of puncta was calculated for each time point and normalized to the ratio at time 0 (6 hours posttransfection).

### Cell viability assay

Cell viability was assessed using the Cell Counting Kit 8 (WST-8/CCK8) (Abcam, ab228554). Briefly, PK13 cells were seeded at a density of 6000 cells per well in 96-well plates (Costar) and allowed to adhere overnight at 37°C. On the following day, the medium was replaced with 100 μl of fresh culture medium containing varying concentrations of CK-666. Cells were then incubated at 37°C for the specified time. After incubation, 10 μl of WST-8 reagent was added to each well, and plates were further incubated at 37°C for 1 hour. Fluorescence was measured at 460 nm using a TECAN Spark 10 M fluorescent plate reader to quantify cell viability.

### Statistical analysis

Statistical analyses were performed using GraphPad Prism. The sample size, number of biological replicates, and statistical tests used for each analysis are specified in the figure legends.

## References

[1] D. M. Knipe, P. M. Howley, *Fields’ Virology* (Lippincott Williams & Wilkins, 2007).

[2] B. Rima, A. Balkema-Buschmann, W. G. Dundon, P. Duprex, A. Easton, R. Fouchier, G. Kurath, R. Lamb, B. Lee, P. Rota, L. Wang, Ictv Report Consortium, ICTV virus taxonomy profile: *Paramyxoviridae*. J. Gen. Virol. 100, 1593–1594 (2019). 31609197 10.1099/jgv.0.001328 PMC7273325

[3] A. Portnoy, M. Jit, M. Ferrari, M. Hanson, L. Brenzel, S. Verguet, Estimates of case-fatality ratios of measles in low-income and middle-income countries: A systematic review and modelling analysis. Lancet Glob. Health 7, e472–e481 (2019). 30797735 10.1016/S2214-109X(18)30537-0 PMC6418190

[4] G. R. Abedi, M. M. Prill, G. E. Langley, M. E. Wikswo, G. A. Weinberg, A. T. Curns, E. Schneider, Estimates of parainfluenza virus-associated hospitalizations and cost among children aged less than 5 years in the United States, 1998-2010. J. Pediatric Infect. Dis. Soc. 5, 7–13 (2016). 26908486 10.1093/jpids/piu047 PMC5813689

[5] Aditi, M. Shariff, Nipah virus infection: A review. Epidemiol. Infect. 147, e95 (2019). 30869046 10.1017/S0950268819000086 PMC6518547

[6] M. Amaya, C. C. Broder, Vaccines to emerging viruses: Nipah and Hendra. Annu. Rev. Virol. 7, 447–473 (2020). 32991264 10.1146/annurev-virology-021920-113833 PMC8782152

[7] A. J. Battisti, G. Meng, D. C. Winkler, L. W. McGinnes, P. Plevka, A. C. Steven, T. G. Morrison, M. G. Rossmann, Structure and assembly of a paramyxovirus matrix protein. Proc. Natl. Acad. Sci. U.S.A. 109, 13996–14000 (2012). 22891297 10.1073/pnas.1210275109 PMC3435216

[8] Z. Ke, J. D. Strauss, C. M. Hampton, M. A. Brindley, R. S. Dillard, F. Leon, K. M. Lamb, R. K. Plemper, E. R. Wright, Promotion of virus assembly and organization by the measles virus matrix protein. Nat. Commun. 9, 1736 (2018). 29712906 10.1038/s41467-018-04058-2 PMC5928126

[9] L. Liljeroos, J. T. Huiskonen, A. Ora, P. Susi, S. J. Butcher, Electron cryotomography of measles virus reveals how matrix protein coats the ribonucleocapsid within intact virions. Proc. Natl. Acad. Sci. U.S.A. 108, 18085–18090 (2011). 22025713 10.1073/pnas.1105770108 PMC3207687

[10] M. J. Norris, M. L. Husby, W. B. Kiosses, J. Yin, R. Saxena, L. J. Rennick, A. Heiner, S. S. Harkins, R. Pokhrel, S. L. Schendel, K. M. Hastie, S. Landeras-Bueno, Z. L. Salie, B. Lee, P. P. Chapagain, A. Maisner, W. P. Duprex, R. V. Stahelin, E. O. Saphire, Measles and Nipah virus assembly: Specific lipid binding drives matrix polymerization. Sci. Adv. 8, eabn1440 (2022). 35857835 10.1126/sciadv.abn1440 PMC9299542

[11] T. M. Svitkina, Actin cell cortex: Structure and molecular organization. Trends Cell Biol. 30, 556–565 (2020). 32278656 10.1016/j.tcb.2020.03.005 PMC7566779

[12] D. Walsh, M. H. Naghavi, Exploitation of cytoskeletal networks during early viral infection. Trends Microbiol. 27, 39–50 (2019). 30033343 10.1016/j.tim.2018.06.008 PMC6309480

[13] S. Bedi, A. Ono, Friend or foe: The role of the cytoskeleton in influenza a virus assembly. Viruses 11, 46 (2019). 30634554 10.3390/v11010046 PMC6356976

[14] T. Serrano, S. Frémont, A. Echard, Get in and get out: Remodeling of the cellular actin cytoskeleton upon HIV-1 infection. Biol. Cell 115, 2200085 (2023). 10.1111/boc.202200085 36597754

[15] M. P. Taylor, O. O. Koyuncu, L. W. Enquist, Subversion of the actin cytoskeleton during viral infection. Nat. Rev. Microbiol. 9, 427–439 (2011). 21522191 10.1038/nrmicro2574 PMC3229036

[16] E. Dietzel, L. Kolesnikova, B. Sawatsky, A. Heiner, M. Weis, G. P. Kobinger, S. Becker, V. von Messling, A. Maisner, Nipah virus matrix protein influences fusogenicity and is essential for particle infectivity and stability. J. Virol. 90, 2514–2522 (2016). 10.1128/JVI.02920-15 PMC4810686 26676785

[17] W. Bohn, G. Rutter, H. Hohenberg, K. Mannweiler, P. Nobis, Involvement of actin filaments in budding of measles virus: Studies on cytoskeletons of infected cells. Virology 149, 91–106 (1986). 3946081 10.1016/0042-6822(86)90090-5

[18] V. Miazza, G. Mottet-Osman, S. Startchick, C. Chaponnier, L. Roux, *Sendai virus* induced cytoplasmic actin remodeling correlates with efficient virus particle production. Virology 410, 7–16 (2011). 21075412 10.1016/j.virol.2010.10.003

[19] A. Thomas, C. Mariani-Floderer, M. R. López-Huertas, N. Gros, E. Hamard-Péron, C. Favard, T. Ohlmann, J. Alcamí, D. Muriaux, Involvement of the Rac1-IRSp53-Wave2-Arp2/3 signaling pathway in HIV-1 Gag particle release in CD4 T cells. J. Virol. 89, 8162–8181 (2015). 26018170 10.1128/JVI.00469-15 PMC4524266

[20] J. D. Rotty, C. Wu, J. E. Bear, New insights into the regulation and cellular functions of the ARP2/3 complex. Nat. Rev. Mol. Cell Biol. 14, 7–12 (2013). 23212475 10.1038/nrm3492

[21] B. Ding, H. Y. Narvaez-Ortiz, Y. Singh, G. M. Hocky, S. Chowdhury, B. J. Nolen, Structure of Arp2/3 complex at a branched actin filament junction resolved by single-particle cryo-electron microscopy. Proc. Natl. Acad. Sci. U.S.A. 119, e2202723119 (2022). 35622886 10.1073/pnas.2202723119 PMC9295785

[22] M. Mehedi, T. McCarty, S. E. Martin, C. Le Nouën, E. Buehler, Y.-C. Chen, M. Smelkinson, S. Ganesan, E. R. Fischer, L. G. Brock, B. Liang, S. Munir, P. L. Collins, U. J. Buchholz, Actin-Related Protein 2 (ARP2) and virus-induced filopodia facilitate human respiratory syncytial virus spread. PLOS Pathog. 12, e1006062 (2016). 27926942 10.1371/journal.ppat.1006062 PMC5142808

[23] R. Dibsy, E. Bremaud, J. Mak, C. Favard, D. Muriaux, HIV-1 diverts cortical actin for particle assembly and release. Nat. Commun. 14, 6945 (2023). 37907528 10.1038/s41467-023-41940-0 PMC10618566

[24] M. Pentecost, A. A. Vashisht, T. Lester, T. Voros, S. M. Beaty, A. Park, Y. E. Wang, T. E. Yun, A. N. Freiberg, J. A. Wohlschlegel, B. Lee, Evidence for ubiquitin-regulated nuclear and subnuclear trafficking among *Paramyxovirinae* matrix proteins. PLOS Pathog. 11, e1004739 (2015). 25782006 10.1371/journal.ppat.1004739 PMC4363627

[25] T. Takimoto, K. G. Murti, T. Bousse, R. A. Scroggs, A. Portner, Role of matrix and fusion proteins in budding of Sendai virus. J. Virol. 75, 11384–11391 (2001). 11689619 10.1128/JVI.75.23.11384-11391.2001 PMC114724

[26] Q. Wang, J. Liu, Y. Luo, V. Kliemke, G. L. Matta, J. Wang, Q. Liu, The nanoscale organization of the Nipah virus fusion protein informs new membrane fusion mechanisms. eLife 13, RP97017 (2024). 10.7554/eLife.97017 PMC11695058 39745781

[27] Q. Liu, L. Chen, H. C. Aguilar, K. C. Chou, A stochastic assembly model for Nipah virus revealed by super-resolution microscopy. Nat. Commun. 9, 3050 (2018). 30076303 10.1038/s41467-018-05480-2 PMC6076310

[28] S. V. Pageon, T. Tabarin, Y. Yamamoto, Y. Ma, P. R. Nicovich, J. S. Bridgeman, A. Cohnen, C. Benzing, Y. Gao, M. D. Crowther, K. Tungatt, G. Dolton, A. K. Sewell, D. A. Price, O. Acuto, R. G. Parton, J. J. Gooding, J. Rossy, J. Rossjohn, K. Gaus, Functional role of T-cell receptor nanoclusters in signal initiation and antigen discrimination. Proc. Natl. Acad. Sci. U.S.A. 113, E5454–E5463 (2016). 27573839 10.1073/pnas.1607436113 PMC5027455

[29] Q. T. Liu, Q. Wang, Y. Zhang, V. Kliemke, Q. Liu, K. C. Chou, The nanoscale organization of Nipah virus matrix protein revealed by super-resolution microscopy. Biophys. J. 121, 2290–2296 (2022). 35614854 10.1016/j.bpj.2022.05.026 PMC9279348

[30] S. Malkusch, U. Endesfelder, J. Mondry, M. Gelléri, P. J. Verveer, M. Heilemann, Coordinate-based colocalization analysis of single-molecule localization microscopy data. Histochem. Cell Biol. 137, 1–10 (2012). 22086768 10.1007/s00418-011-0880-5

[31] E. Hunter, “Virus assembly” in *Fields’ Virology* (Lippincott Williams & Wilkins, 2007), pp. 141–168.

[32] G. P. Johnston, B. Bradel-Tretheway, P. D. Piehowski, H. M. Brewer, B. N. R. Lee, N. T. Usher, J. L. R. Zamora, V. Ortega, E. M. Contreras, J. R. Teuton, J. P. Wendler, K. M. Matz, J. N. Adkins, H. C. Aguilar, Nipah virus-like particle egress is modulated by cytoskeletal and vesicular trafficking pathways: A validated particle proteomics analysis. mSystems 4, e00194-19 (2019). 31551400 10.1128/mSystems.00194-19 PMC6759566

[33] N. M. Vera-Velasco, M. J. García-Murria, M. M. Sánchez Del Pino, I. Mingarro, L. Martinez-Gil, Proteomic composition of Nipah virus-like particles. J. Proteomics 172, 190–200 (2018). 29092793 10.1016/j.jprot.2017.10.012

[34] R. M. Giuffre, D. R. Tovell, C. M. Kay, D. L. Tyrrell, Evidence for an interaction between the membrane protein of a paramyxovirus and actin. J. Virol. 42, 963–968 (1982). 6285006 10.1128/jvi.42.3.963-968.1982 PMC256929

[35] K. C. Stallcup, C. S. Raine, B. N. Fields, Cytochalasin B inhibits the maturation of measles virus. Virology 124, 59–74 (1983). 6681685 10.1016/0042-6822(83)90290-8

[36] T. Serrano, N. Casartelli, F. Ghasemi, H. Wioland, F. Cuvelier, A. Salles, M. Moya-Nilges, L. Welker, S. Bernacchi, M. Ruff, A. Jégou, G. Romet-Lemonne, O. Schwartz, S. Frémont, A. Echard, HIV-1 budding requires cortical actin disassembly by the oxidoreductase MICAL1. Proc. Natl. Acad. Sci. U.S.A. 121, e2407835121 (2024). 39556735 10.1073/pnas.2407835121 PMC11621841

[37] C. Lacouture, B. Carrio, C. Favard, D. Muriaux, HIV-1 assembly – when virology meets biophysics. J. Cell Sci. 137, jcs262064 (2024). 39404604 10.1242/jcs.262064

[38] Y. E. Wang, A. Park, M. Lake, M. Pentecost, B. Torres, T. E. Yun, M. C. Wolf, M. R. Holbrook, A. N. Freiberg, B. Lee, Ubiquitin-regulated nuclear-cytoplasmic trafficking of the Nipah virus matrix protein is important for viral budding. PLOS Pathog. 6, e1001186 (2010). 21085610 10.1371/journal.ppat.1001186 PMC2978725

[39] B. Huang, H. Babcock, X. Zhuang, Breaking the diffraction barrier: Super-resolution imaging of cells. Cell 143, 1047–1058 (2010). 21168201 10.1016/j.cell.2010.12.002 PMC3272504

[40] S. V. Pageon, P. R. Nicovich, M. Mollazade, T. Tabarin, K. Gaus, Clus-DoC: A combined cluster detection and colocalization analysis for single-molecule localization microscopy data. Mol. Biol. Cell 27, 3627–3636 (2016). 27582387 10.1091/mbc.E16-07-0478 PMC5221594

[41] S. Shubhrasmita Sahu, P. Sarkar, A. Chattopadhyay, Quantitation of F-actin in cytoskeletal reorganization: Context, methodology and implications. Methods 230, 44–58 (2024). 39074540 10.1016/j.ymeth.2024.07.009

[42] S. Choi, J. Kelber, X. Jiang, J. Strnadel, K. Fujimura, M. Pasillas, J. Coppinger, R. Klemke, Procedures for the biochemical enrichment and proteomic analysis of the cytoskeletome. Anal. Biochem. 446, 102–107 (2014). 24161902 10.1016/j.ab.2013.10.025 PMC3947189

