# Supplementary material for: Nipah virus matrix protein uses cortical actin to stabilize the virus assembly sites and promote budding
Source: Sci Adv. 2025 Sep 19;11(38):eadw4609. doi: 10.1126/sciadv.adw4609 (PMC12448087; doi:10.1126/sciadv.adw4609)
Supplement: Supplementary file 1 — Figs. S1 to S6 [file sciadv.adw4609_sm.pdf]

Science Advances
AAAS

Supplementary Materials for

Nipah virus matrix protein uses cortical actin to stabilize the virus assembly sites and promote budding

Jingjing Wang *et al.*

Corresponding author: Qian Liu, qian.liu3@mcgill.ca

Sci. Adv. **11**, eadw4609 (2025)
DOI: 10.1126/sciadv.adw4609

This PDF file includes:

Figs. S1 to S6

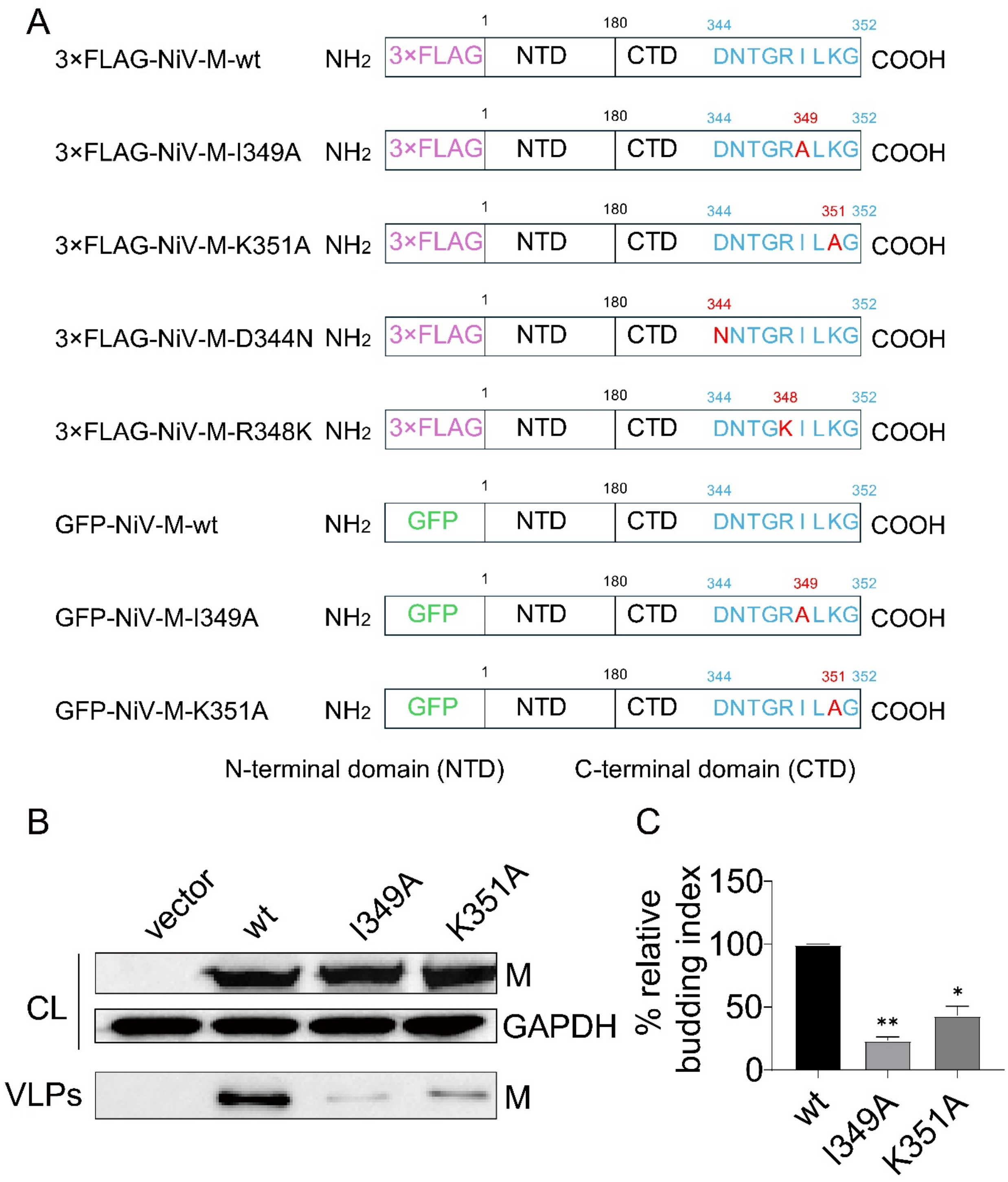

Fig. S1- related to Fig. 1. NiV-M constructs and VLP production of GFP-tagged NiV-M constructs. (**A**) A diagram showing the GFP and 3×FLAG-tagged NiV-M constructs. (**B**) VLPs and cell lysates harvested from 293T cells transfected with empty pcDNA3 vector (vector) and plasmids coding for GFP-tagged wt, I349A, and K351A were analyzed using western blot. NiV-M was detected using a goat anti-GFP antibody. (**C**) Relative budding index is determined based on integrated immunoblot density in (**B**). Bars represent mean ± SEM. *p* value was

obtained using one-way ANOVA with post-hoc correction. *: $p \leq 0.05$, **: $p \leq 0.01$. Results from ≥ 3 independent experiments are shown.

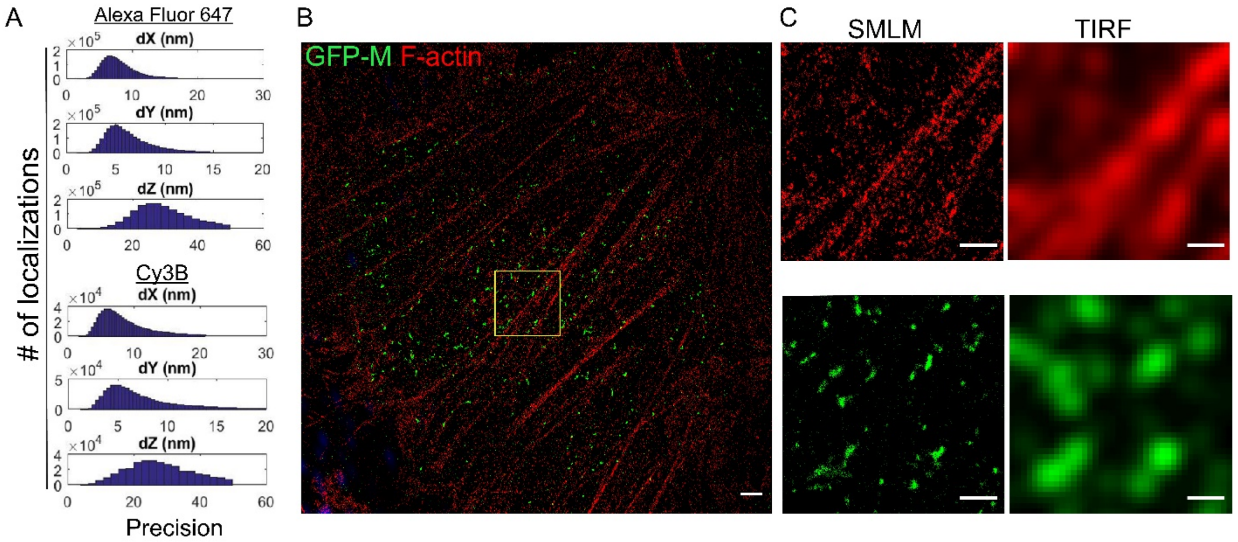

Fig. S2-related to Fig. 2. The localization precision of SMLM. (**A**) The localization precision of SMLM for AlexaFluor 647 (red) and cy3B (green). (**B**) x-y cross section (600 nm in z) of GFP-NiV-M (green) and F-actin (red) at the ventral membrane of PK13 cells. SMLM images were taken at the cell-cover glass interface using total Internal Reflection (TIRF) illumination. Scale bar: 2 µm. (**C**) The boxed regions are enlarged to show detailed structures in both channels in SMLM and TIRF microscopy. Scale bar: 1 µm.

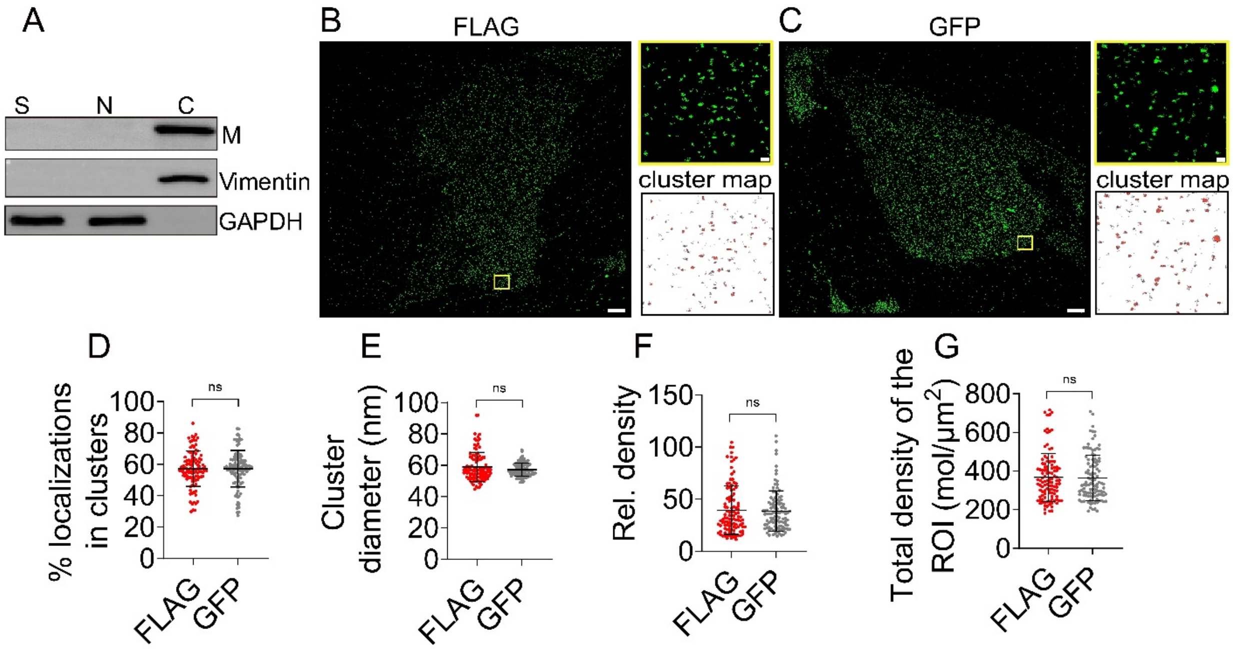

Fig. S3-related to Fig. 2. NiV-M is associated with the host cytoskeleton and its clustering pattern remains similar regardless of the labeling tags. (**A**) PK13 cells were transfected with 3×FLAG-NiV-M for 48 hrs and the soluble compartment (S), nuclear compartment (N), and cytoskeleton (C) were extracted. The partitioning of NiV-M to each compartment was analyzed by western blot analysis. GAPDH is a marker for S and N compartments, and vimentin C compartment. NiV-M was detected using a mouse anti-FLAG antibody, GAPDH a mouse anti-GAPDH antibody, and vimentin a rabbit anti-vimentin antibody. (**B-G**) PK13 cells expressing 3×FLAG-NiV-M or GFP-NiV-M were subjected to SMLM imaging. (**B, C**) x-y cross-section (600 nm in z) of the SMLM images of 3×FLAG-NiV-M (**B**) and GFP-NiV-M (**C**) at the ventral membrane of PK13 cells. The boxed regions are enlarged, and the cluster maps of the NiV-M localizations are shown. Scale bar: 1 μm and 200 nm. The percentage of localizations in clusters (**D**), diameter (**E**), relative density (**F**), and total density of the ROI (mol/μm^2) (**G**) of 3×FLAG-NiV-M (n = 108) and GFP-NiV-M (n = 108) from 10-20 cells per group. Bars represent mean ± SD. p value was obtained using Student's t-test with Welch correction. ns: $p > 0.05$. Results from ≥ 3 independent experiments are shown.

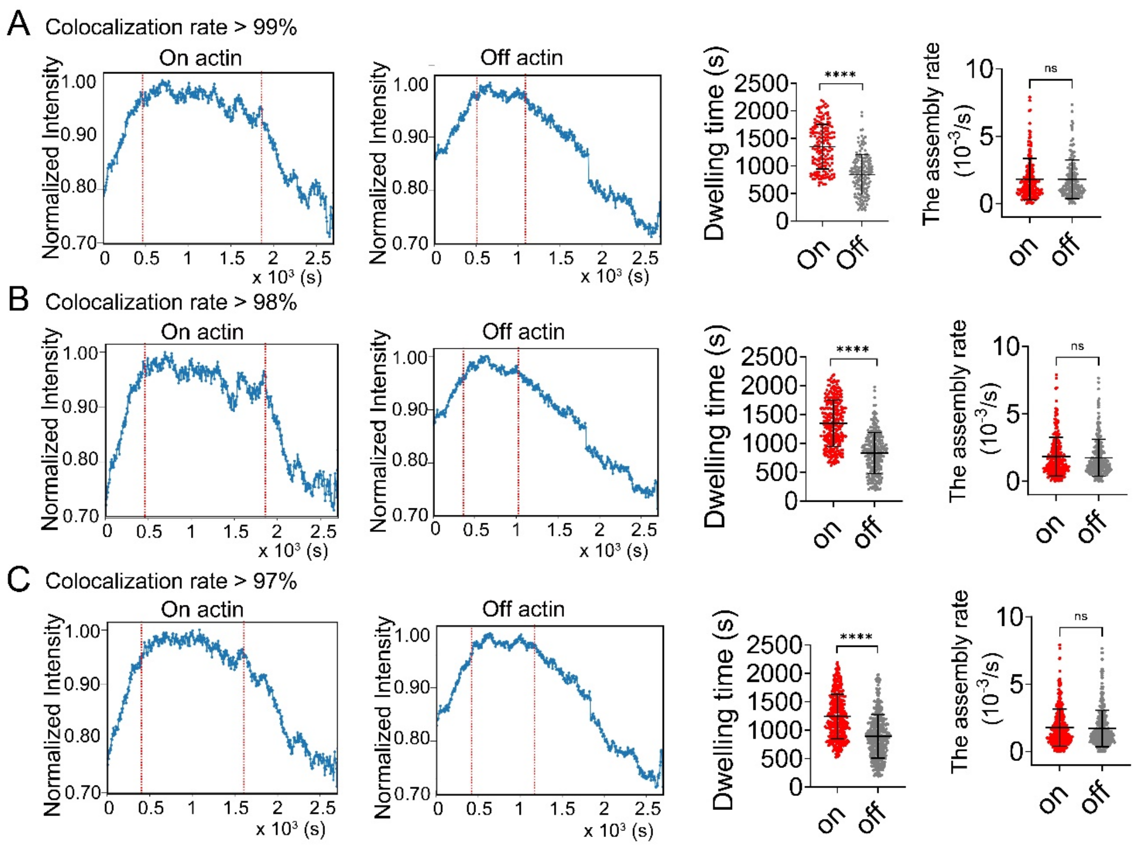

Fig. S4. The differences in the dwelling time and assembly rate between "on actin" and "off actin" tracks remain consistent regardless of the classification thresholds used. Each "on-actin" track has > 99% (**A**), 98% (**B**), or 97% (**C**) of total events co-localizing with F-actin, whereas each "off-actin" track has < 1% (**A**), 2% (**B**), or 3% (**C**) of total events co-localizing with F-actin. The total number of "on actin" and "off actin" tracks is 166 and 155 in (**A**), 260 and 238 in (**B**), and 406 and 391 in (**C**), respectively. The tracks from each group were collected from 30-40 cells. The dwelling time and assembly rate of "on actin" and "off actin" tracks are shown for each threshold. Bars represent mean ± SD. p value was obtained using Student's t-test with Welch correction. ns: $p > 0.05$, ****: $p \leq 0.0001$. The dataset in (B) is the same as that in Fig. 4G-J.

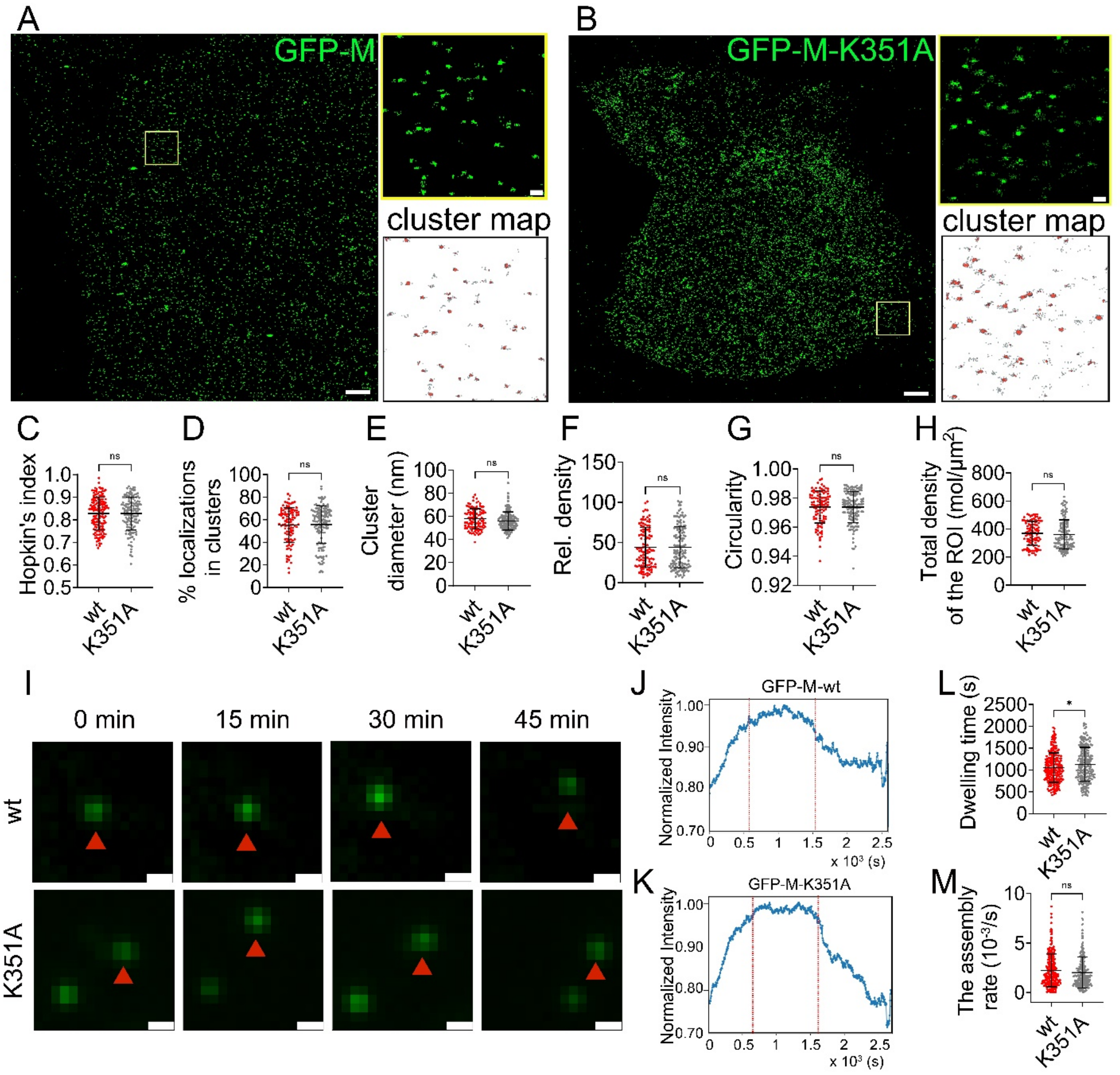

Fig. S5- related to Fig. 5. The K351A mutant at the actin-binding domain did not alter the nano-organization of NiV-M but slightly increased its membrane association. (**A** and **B**) x-y cross-section (600 nm in z) of the SMLM images of GFP-NiV-M-wt (**A**) and K351A (**B**) at the ventral membrane of PK13 cells. The boxed regions are enlarged, and the cluster maps of the NiV-M localizations are shown. Scale bars: 1 μm and 200 nm. (**C**) The Hopkin's index of the localizations of NiV-M-wt and K351A. (**D-H**) The percentage of localizations in clusters (**D**), cluster diameter (**E**), relative density (**F**), circularity (**G**), and total density of the ROI (**H**) of wt and K351A are shown. Sample size n = 105 (wt) and 140 (K351A) from 12-18 cells. (**I**) Representative images of PK13 cells expressing GFP-tagged NiV-M-wt and K351A. Scale bar: 1 μm. (**J** and **K**) The intensity profiles of NiV-M-wt and K351A puncta were averaged from 232 and 254 tracks from 30-40 cells, respectively. Red dashed lines separate three phases. The dwelling time (**L**) and assembly rate (**M**) of NiV-M-wt and K351A tracks. Bars represent mean ± SD. Bars represent mean ± SD. *p* value was obtained using Student's *t*-test with Welch

correction. ns: $p > 0.05$; *: $p \leq 0.05$. Results from ≥ 3 independent experiments are shown. The dataset for wt in (**J-M**) is the same as that in Fig. 5J-M.

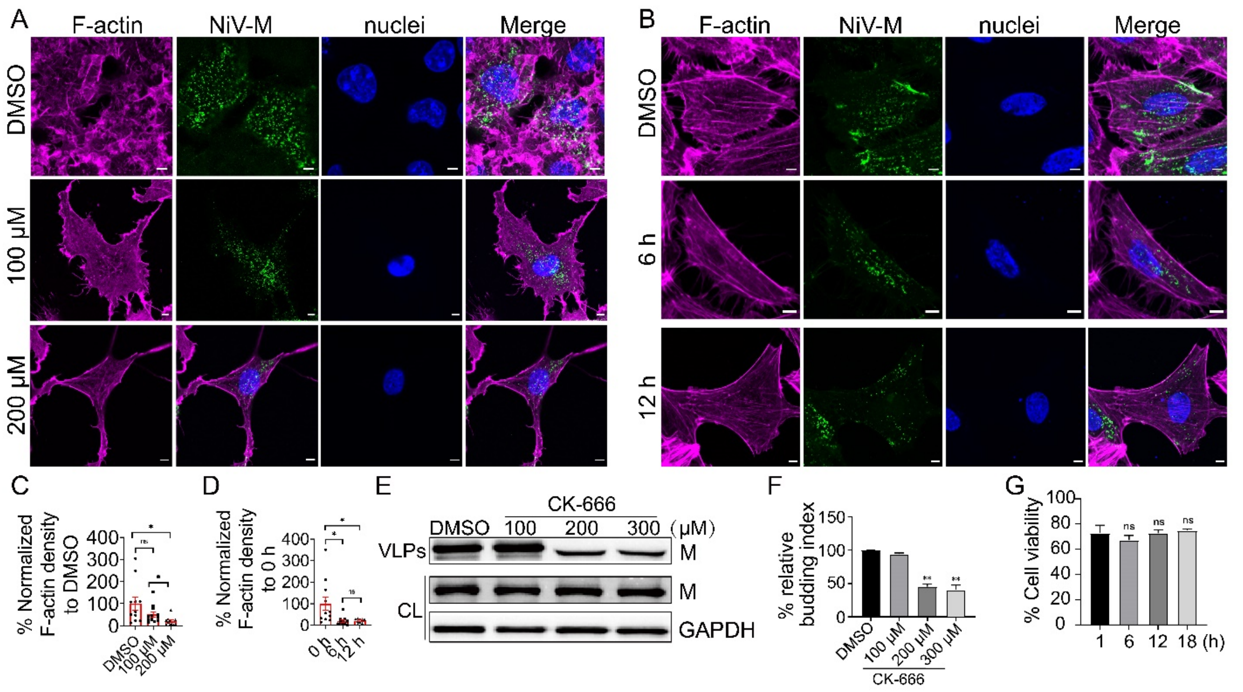

Fig. S6-related to Fig. 7. The effects of CK-666 on F-actin network, NiV VLP production, and cell viability. (**A**) COS-7 cells stably expressing 3×FLAG-NiV-M treated by DMSO, 100 μM, and 200 μM CK-666 for 18 hrs were fixed. NiV-M (green) was detected using a mouse anti-FLAG antibody and donkey anti-goat cy3B antibody. F-actin (magenta) was detected using Alexa Fluor 647 conjugated phalloidin. The cell nucleus was stained using DAPI. Representative single-plane images acquired at a 350 nm z-step interval are shown. Scale bar: 5 μm. (**B**) PK13 cells expressing GFP-NiV-M were treated with DMSO and 200 μM CK-666 at 6 hrs post-transfection. Cells were fixed at 6 hrs and 12 hrs after the addition of CK-666. NiV-M was detected by GFP signal, and F-actin and the cell nucleus were detected as described in (**A**). Representative single-plane images acquired at a 350 nm z-step interval are shown. Scale bar: 5 μm. (**C**) The F-actin density was determined using the total fluorescence intensity of Alexa647-phalloidin bound to F-actin in each cell, and then normalized to that of DMSO. Results are from 11-12 cells. (**D**) The F-actin density was determined as described in (**C**), and normalized to that of 0 hr. Results are from 9-11 cells. (**E**) VLPs and cell lysates were harvested from PK13 cells transfected with 3×FLAG-NiV-M and treated with 300 μM DMSO, 100 μM, 200 μM, and 300 μM CK-666. NiV-M in the cell lysate (CL) and VLPs were analyzed using Western Blot and detected using a mouse anti-FLAG antibody. (**F**) Relative budding index is determined based on integrated immunoblot density in (**E**). Results from ≥ 3 independent experiments are shown. (**G**) PK13 cells were treated with 200 μM CK-666 for 1 h, 6 h, 12 h, and 18 h, respectively. The cell viability was detected using a CCK8 kit. Bars represent mean ± SD (**C** and **D**) mean ± SEM (**F** and **G**). *p* value was obtained using one-way ANOVA with post-hoc correction. ns: $p > 0.05$, *: $p \leq 0.05$, **: $p \leq 0.01$. Results from ≥ 3 independent experiments are shown.
